# Overall proportion of orthorexia nervosa symptoms: A systematic review and meta-analysis including 30 476 individuals from 18 countries

**DOI:** 10.7189/jogh.13.04087

**Published:** 2023-11-03

**Authors:** José Francisco López-Gil, Pedro Juan Tárraga-López, Maria Soledad Hershey, Rubén López-Bueno, Héctor Gutiérrez-Espinoza, Antonio Soler-Marín, Alejandro Fernández-Montero, Desirée Victoria-Montesinos

**Affiliations:** 1 Navarrabiomed, Hospital Universitario de Navarra, Universidad Pública de Navarra, Instituto de Investigación Sanitaria de Navarra, Pamplona, Navarra, Spain; 2 Department of Environmental Health, Harvard University T.H. Chan School of Public Health, Boston, Massachusetts, USA; 3 One Health Research Group, Universidad de Las Américas, Quito, Ecuador; 4 Departamento de Ciencias Médicas, Facultad de Medicina, Universidad Castilla-La Mancha, Albacete, Spain; 5 University of Navarra, Department of Preventive Medicine and Public Health, School of Medicine, Pamplona, Spain; 6 Department of Physical Medicine and Nursing, University of Zaragoza, Zaragoza, Spain; 7 Escuela de Fisioterapia, Universidad de las Américas, Quito, Ecuador; 8 Faculty of Pharmacy and Nutrition, Universidad Católica San Antonio de Murcia, Murcia, Spain; 9 Department of Occupational Medicine, University of Navarra, Instituto de Investigación Sanitaria de Navarra, Pamplona, Spain

## Abstract

**Background:**

To date, no previous meta-analysis has determined the overall proportion of orthorexia nervosa symptoms on a global scale. The aim of the present study was 2-fold: first, to establish the overall proportion of orthorexia nervosa symptoms on a global scale, assessed with the ORTO-15 questionnaire; and second, to determine the role of sex, type of population, mean age, body mass index, and the temporal trend in relation to orthorexia nervosa symptoms.

**Methods:**

Four databases were searched (PubMed, Scopus, Web of Science, and Cochrane Database of Systematic Reviews) with date limits from January 2005 to June 2023. Studies assessing the proportion of orthorexia nervosa assessed using the ORTO-15 questionnaire with a cutoff of <35 or <40 points were included in this review.

**Results:**

The overall proportion of orthorexia nervosa symptoms (using the cutoff <35 points) was 27.5% (95% confidence interval (CI) = 23.5-31.6, *I^2^* = 97.0%). In addition, no significant differences were observed between females (34.6%, 95% CI = 29.5-39.8, *I^2^* = 96.1%) and males (32.1%, 95% CI = 26.5-38.1, *I^2^* = 93.1%). According to the type of population, the highest overall proportion was found in people focused on sports performance or body composition (34.5%, 95% CI = 23.1-47.0, *I^2^* = 98.0%). Notwithstanding, caution should be exercised in interpreting this result, as reverse causality could be a potential pitfall in this relationship.

**Conclusions:**

We found that approximately three out of 10 study participants showed orthorexia nervosa symptoms according to the ORTO-15 tool. This overall proportion was higher in those participants who were athletes or fitness practitioners. Over the years, the proportion of orthorexia nervosa symptoms seems to be increasing. These high percentages and their increase are worrisome from a public health perspective and highlight the need to develop psychometric instruments to aid in clinical diagnosis and treatment efficacy.

**Registration:**

PROSPERO (CRD42022350873).

Eating disorders are severe psychiatric disorders characterised by abnormal eating or weight control behaviours, which can lead to serious health problems [1]. These disorders include anorexia nervosa, bulimia nervosa, binge eating disorder, and eating disorder not otherwise specified (EDNOS), which are defined on the basis of individual signs and symptoms with degrees of severity described in the Diagnostic and Statistical Manual of Mental Disorders-version 5 (DSM-5) [2]. Among EDNOS, orthorexia nervosa (ON) has raised the most public awareness; however, it is one of the most controversial medical conditions [3]. In 1997, ON was conceived as a disorder not related to concerns about weight and shape but rather motivated exclusively by the desire to eat healthy food [4]. Nonetheless, this same author recently acknowledged that individuals’ perception of “healthy” eating could focus on foods and eating patterns that promote thinness and weight loss, thus implying that ON could involve motivation related to weight and/or shape concerns [5]. At present, there is no universally shared definition of ON, the diagnostic criteria are under debate, and the psychometric properties of the tools used across the scientific literature revealed certain methodological flaws [6].

Despite the above, the pathological aspect of ON could be conceptualised as an excessive fixation on consuming foods believed to be healthy [5]. Individuals with ON are primarily concerned with the quality rather than the quantity of food they consume [7]. They invest significant time and effort into examining the origin (such as checking for pesticide exposure in vegetables or whether dairy products come from hormone-supplemented cows), processing (including assessing potential nutrient loss during cooking or the addition of micronutrients, artificial flavouring, or preservatives), and packaging (such as evaluating if the food may contain carcinogenic compounds derived from plastic or if labels provide sufficient information about ingredient quality) of food available for purchase in the market [8]. This obsession with food dictates their lives, resulting in difficulties in social interactions, family relationships, and work performance [9].

The exact cause of ON is not fully understood, but it is believed to involve multiple factors. People with ON experience neurocognitive challenges that resemble those found in individuals with anorexia nervosa and obsessive-compulsive disorder, which include difficulties with flexible problem-solving, external attention, and working memory [7,10]. Due to the shared cognitive impairments and similar symptoms, it is possible that individuals with ON, anorexia nervosa, and obsessive-compulsive disorder exhibit comparable brain dysfunction [11]. Several factors play a role in the development of both anorexia nervosa and potentially ON. These factors encompass the formation of food preferences, inherited variances in taste perception, food neophobia or selectivity, having overweight or obesity, parental feeding practices, and a history of parental eating [12]. Additionally, characteristics such as perfectionism, emphasis on appearance, preoccupation with weight, self-perceived weight classification, attachment styles characterised by fear or dismissal, appearance orientation, and a history of an eating disorder have also been identified [13].

Regarding the proportion of ON, some previous systematic reviews have tried to determine the proportion of this type of eating disorders [14-16]. For instance, Niedzielski et al. [14] found that the proportions of ON symptoms in the general population (assessed by the ORTO-15 questionnaire) ranged from 6.9% to 75.2% (90.6% in some specific groups). However, to our knowledge, no previous meta-analysis has determined the overall proportion of ON symptoms on a global scale (using the ORTO-15 questionnaire). Given the high disparity and inconsistency in the proportions found in previous systematic reviews using different cutoff points, it seems necessary to establish an overall proportion using a harmonised methodology (e.g. similar cutoff points). Similarly, from an epidemiological point of view, it has been suggested that ON must be treated as a stand-alone eating disorder and included as an emerging condition in the DSM classification [17].

Thus, identifying the magnitude of ON symptoms and their distribution among at-risk populations may be crucial for planning and executing public health initiatives aimed at preventing, detecting, and managing this disorder, given that traditional treatment approaches for eating disorders (e.g. anorexia nervosa) may not be appropriate for those with ON symptoms [3]. Therefore, the aim of the present study was 2-fold: first, to establish the overall proportion of ON symptoms on a global scale, assessed with the ORTO-15 questionnaire, one of the most widely used methods to study ON symptoms [18]; and second, to determine the role of sex, type of population, mean age, body mass index, and the temporal trend in relation to ON symptoms.

## METHODS

This systematic review and meta-analysis was registered in the International prospective register of systematic reviews (PROSPERO) (registration number: CRD42022350873) and conducted according to the Preferred reporting items for systematic reviews and meta-analyses – PRISMA statement [19].

### Eligibility criteria

Based on the outcome, we included studies that reported the prevalence/proportion of ON symptoms (i.e. less than 35 or 40 points in the ORTO-15 questionnaire) [20,21]. Regarding the study design, we had no restrictions except for systematic reviews and/or meta-analyses and qualitative and case studies.

The exclusion criteria included: studies conducted exclusively among people with other eating disorders (e.g. anorexia) or who had a diagnosis of physical or mental disorders, studies that were published before 2005 since the ORTO-15 questionnaire was designed in that year [20], studies based on data from the same surveys/studies to avoid duplication, and qualitative and case studies.

### Information sources and search strategy

Two researchers (JFLG and DVM) systematically searched PubMed, Scopus, Web of Science, and Cochrane Database of Systematic Reviews databases, with date limits from January 2005 to June 2023. Studies were identified with the following search terms: “orthorexia”, “orthorexia nervosa”, “orthorexic behaviours”, and ORTO-15. The search terms were adapted for each database in combination with database-specific filters (provided in Table S1 in the **Online Supplementary Document**). In addition, the list of references of the studies included in this review and in previous systematic reviews [14,15,22,23] were thoroughly reviewed to ensure that no eligible studies were excluded.

### Selection process

After identifying eligible studies, Mendeley (Version for Windows 10; Elsevier, Amsterdam, the Netherlands) was used to remove duplicate studies. Two members of the research team (JFLG and DVM) conducted the selection process independently and screened all titles and abstracts to identify potentially relevant articles for further review in the full-text phase. A third researcher (PJTL) participated to resolve any discrepancies.

### Data items

The proportion of participants with ON symptoms was extracted by one researcher (DVM), while another researcher (JFLG) checked the data for accuracy. In case of a discrepancy between these two researchers, a third researcher (PJTL) reviewed the information.

### Risk of bias

Two researchers (DVM and JFLG) independently assessed the risk of study bias in the included studies. This assessment was performed using a specific tool for prevalence/proportion studies [24]. The tool consists of 10 items that address both the external and internal validity of prevalence/proportion studies. Each item can be classified as “yes (low risk)” or “no (high risk)”, which are assigned zero and one point, respectively. The overall risk of study bias is deemed to be at “low risk of bias”, “moderate risk of bias” or “high risk of bias” if the total points scored are 0-3, 4-6, or 7-9, respectively.

### Small-study effects and publication bias

Small-study effects and publication bias were examined using the Doi plot and the Luis Furuya-Kanamori (LFK) index [25]. No asymmetry, minor asymmetry, or major asymmetry were considered with values of <-2, between -2 and -1, and >-1, respectively [25].

### Outcome measures

The proportion of ON symptoms (using a cutoff <35 points) was computed based on the raw numerators and denominators found among the studies. We used this more conservative cutoff point (instead of a cutoff <40 points) because many researchers have argued a lack of specificity in identifying those without ON symptoms (i.e. false positives) [21]. However, to offer a wider synthesis of the information, the proportion of ON symptoms using the cutoff <40 points has been reported as supplementary material.

### Statistical analyses

A random-effects model was applied to pool the data using the software R (Version 4.3.0) (R Core Team, Vienna, Austria) and RStudio (Version 2023.06.0 + 421) (Posit, Boston, Massachusetts, USA). The meta package [26] was used to conduct a meta-analysis of single proportions (i.e. metaprop). The pooling of studies was displayed as forest plots using the inverse variance method [27]. To estimate the between-study variance, the restricted maximum likelihood (REML) method was applied. Furthermore, to determine the 95% confidence intervals (CI) for proportions from the selected individual studies, the exact or Clopper-Pearson interval method (i.e. binomial interval method) was utilised. To make the normal distribution assumptions more applicable to significance testing, the Freeman-Tukey double arcsine transformation of proportions was applied. For both the computation of individual study outcomes along with their confidence intervals and for carrying out a meta-analysis, a continuity correction of 0.5 was applied. Intragroup heterogeneity of pooled proportions was also calculated using the heterogeneity statistic (*I*^2^) and its *P*-value.

Subgroup analyses were conducted by sex (male sex or female sex), type of population study (general population, people focused on sports performance or body composition, people from health-related programs or professions, people living with the disease, or people with a special diet), and period of data collection (prior to 2016, between 2016 and 2019, or between 2020 and 2023) using Cochran’s *Q*-test [28]. Only studies that provided information based on these parameters were included in these analyses. When data collection was carried out over a period of time, the end date of data collection was considered. In addition, when the year of data collection was not specified, the year of approval of the ethics committee was considered. Finally, if this date was not indicated, then the publication date was considered.

On the other hand, random-effects meta-regression analyses using the method of moments were estimated to independently assess whether disordered eating symptoms differed by mean age, body mass index, or data collection date (all as continuous variables). This decision was based on a previous review of psychosocial factors related to ON [21]. Finally, a *P*-value <0.05 was considered statistically significant.

## RESULTS

### Study selection

**Figure 1:**
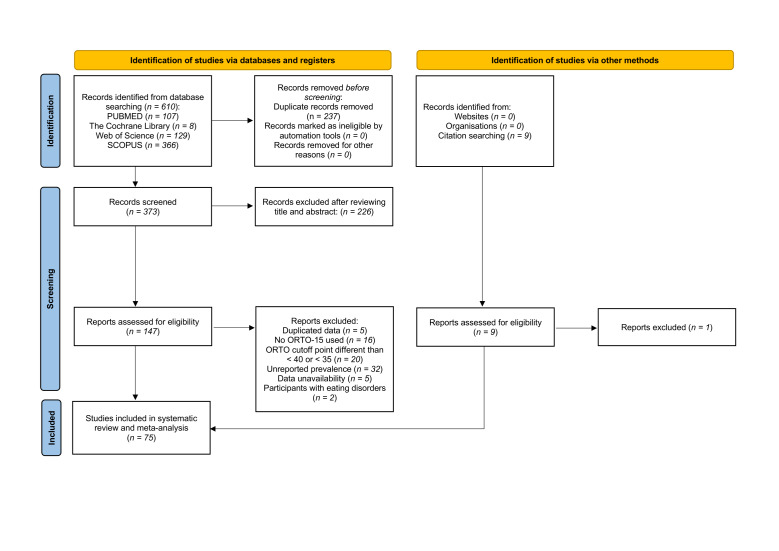
Preferred reporting items for systematic reviews and meta-analyses (PRISMA) flow diagram.

A total of 610 records were identified through database searches (**Figure 1**). After screening for duplicates, 373 records remained. Finally, 147 studies were obtained for full-text review. Of those studies, 80 were excluded for different reasons (Table S2 in the **Online Supplementary Document**). Finally, 75 studies were included in this systematic review (94.7% agreement between reviewers), and all studies were included in the different meta-analyses.

### Study characteristics

**Table 1** summarises the main characteristics of the 75 included studies. A total of 30 476 participants (61.2% women) aged 13-93 years were included in this systematic review and meta-analysis. Segmented information about the studies using the <35 or <40 cutoff point can be found in Tables S3 and S4 in the **Online Supplementary Document**, respectively.

**Table 1:** Characteristics of the studies included (n = 75)

Reference	Year	Country	Study design	Type of population*	Total (n)	Girls (%)	Age (years)	BMI (kilogramme/square metre
Abdullah et al. [29]	2020	Jordan	Cross-sectional	A	421	69.8	23.9	Not reported
Aiello et al. [30]	2022	Spain and Italy	Cross-sectional	C	160	57.5	24.1	22.8
Aksoydan and Camci [31]	2009	Turkey	Cross-sectional	B	94	58.5	33.2	21.8
Albery et al. [32]	2020	United Kingdom	Experimental study	C	80	75.0	29.4	Not reported
Almeida et al. [33]	2018	Portugal	Cross-sectional	B	193	58.5	32.8	Not reported
Asil and Sürücüoğlu [34]	2015	Turkey	Cross-sectional	A	117	86.3	34.0	22.9
Athanasaki et al. [35]	2023	Greece	Cross-sectional	B	96	95.8	23.4	20.2
Bergonzi and Massarollo [36]	2022	Brazil	Cross-sectional	C	61	95.1	23.0	22.2
Bosi et al. [37]	2007	Turkey	Cross-sectional	A	315	53.1	27.2	Not reported
Bert et al. [38]	2019	Italy	Cross-sectional	B	549	25.3	26.7	Not reported
Bién and Pieczykolan [39]	2017	Poland	Cross-sectional	C	280	100	Not reported	Not reported
Bo et al. [40]	2014	Italy	Cross-sectional	A	440	54.1	19.8	16.9
Carpita et al. [41]	2021	Italy	Cross-sectional	D	2426	65.5	26.9	Not reported
Clifford and Blyth [42]	2019	United Kingdom	Case-control	B	215	65.6	21.0	Not reported
Cosentino et al. [43]	2023	Italy	Cross-sectional	A	88	Not reported	40.0	23.7
De Marchi and Baratto [44]	2018	Brazil	Cross-sectional	A	82	93.9	21.0	Not reported
de Souza and Rodrigues [45]	2014	Brazil	Cross-sectional	A	150	100	23.2	Not reported
Dell’Osso et al. [46]	2016	Italy	Cross-sectional	C	2826	40.6	28.9	22.6
Demir and Bayram [47]	2022	Turkey	Cross-sectional	A	310	65.8	31.8	24.2
Demirer and Yardımcı [48]	2023	Turkey	Cross-sectional	C	197	53.3	30.6	23.8
Donini et al. [20]	2005	Italy	Cross-sectional	C	514	Not reported	Not reported	Not reported
Dunn et al. [49]	2017	USA	Cross-sectional	C	275	66.0	21.7	Not reported
Elias et al. [50]	2022	Brazil	Cross-sectional	C	246	43.1	Not reported	Not reported
Erdogan et al. [51]	2022	Turkey	Cross-sectional	E	159	100	33.2	Not reported
Ermumcu and Tek [52]	2018	Turkey	Cross-sectional	C	132	100	31.7	23.6
Farchakh et al. [53]	2019	Lebanon	Cross-sectional	A	627	50.4	21.8	23.4
Freire et al. [54]	2020	Brazil	Cross-sectional	B	60	63.3	26.6	Not reported
Gorrasi et al. [55]	2020	Italy	Cross-sectional	C	918	54.8	20.2	21.9
Gonidakis et al. [56]	2021	Greece	Cross-sectional	C	120	Not reported	Not reported	22.2
Grajek et al. [57]	2022	Poland	Cross-sectional	A	290	60.0	26.0	Not reported
Gubiec et al. [58]	2015	Poland	Cross-sectional	A	155	90.3	22.1	Not reported
Guglielmetti et al. [59]	2022	Italy	Cross-sectional	A	671	53.9	21.0	21.77
Gwioździk et al. [60]	2022	Poland	Cross-sectional	E	420	100	24.0	Not reported
Haddad et al. [61]	2019	Lebanon	Cross-sectional	C	589	66.5	27.6	24.4
Hamid et al. [62]	2018	Malaysia	Cross-sectional	A	138	88.4	21.8	21.4
Hayles et al. [63]	2017	USA	Cross-sectional	C	404	82.7	20.7	Not reported
Heiss et al. [64]	2019	Not reported	Cross-sectional	E	381	80.8	31.0	24.2
Herranz Valera et al. [65]	2014	Spain	Cross-sectional	B	136	65.4	36.7	21.4
Hyrnik et al. [66]	2016	Poland	Cross-sectional	C	1899	52.2	17.4	21.7
Jerez et al. [67]	2015	Chile	Cross-sectional	C	210	45.9	16.7	Not reported
Karadağ et al. [68]	2016	Turkey	Cross-sectional	C	750	50.0	25.8	23.3
Karniej et al. [69]	2023	Poland and Spain	Cross-sectional	C	394	0	38.1	26.1
Koven and Senbonmatsu [10]	2013	USA	Cross-sectional	C	100	79.0	19.3	Not reported
Kujawowicz et al. [70]	2022	Poland	Cross-sectional	D	123	100	34.0	21.4
Labossière and Thibault [71]	2019	Canada	Cross-sectional	B	133	71.4	21.3	Not reported
Lemos et al. [72]	2018	Brazil	Cross-sectional	A	95	100	20.5	21.4
Lorenzon et al. [73]	2020	Brazil	Cross-sectional	B	430	56.7	35.3	Not reported
Łucka et al. [74]	2019	Poland	Cross-sectional	C	864	69.3	19.8	22.6
Maghetti et al. [75]	2015	Italy	Cross-sectional	A	517	53.8	Not reported	23.1
Malmborg et al. [76]	2017	Sweden	Cross-sectional	A	207	56.5	22.8	Not reported
Martinovic et al. [77]	2022	Croatia	Cross-sectional	B	300	45.0	22.4	24.2
Mitrofanova et al. [78]	2021	Greece	Cross-sectional	C	10	80.0	28.4	21.2
Mitrofanova et al. [79]	2021	United Kingdom	Cross-sectional	C	50	60.0	34.0	25.3
Penaforte et al. [80]	2017	Brazil	Cross-sectional	A	141	90.8	21.5	22.9
Plichta and Jezewska-Zychowicz [81]	2019	Poland	Cross-sectional	A	1120	70.4	21.4	22.0
Raguzzini et al. [82]	2021	Italy	Cross-sectional	D	56	51.8	61.7	25.0
Ramacciotti et al. [83]	2011	Italy	Cross-sectional	C	177	63.8	38.0	23.3
Reynolds [84]	2018	Australia	Cross-sectional	C	92	79.3	24.6	Not reported
Rizzieri et al. [85]	2019	Brazil	Cross-sectional	B	65	55.4	29.9	Not reported
Sampaio et al. [86]	2022	Brazil	Cross-sectional	C	150	74.7	Not reported	Not reported
Sanlier et al. [87]	2016	Turkey	Cross-sectional	C	900	58.0	20.4	22.1
Stochel et al. [88]	2015	Poland	Cross-sectional	C	399	53.4	16.9	21.0
Sünbül and Bayrak [89]	2021	Turkey	Cross-sectional	C	580	43.1	20.9	23.2
Surała et al. [90]	2020	Not reported	Cross-sectional	B	273	45.8	20.9	22.2
Tocchetto et al. [91]	2018	Brazil	Cross-sectional	B	50	46.0	23.5	19.7
Turner and Lefevre [92]	2017	Not reported	Cross-sectional	C	680	100	24.7	22.1
Uriegas et al. [93]	2021	USA	Cross-sectional	B	1090	69.4	19.6	23.2
Vaccari et al. [94]	2021	Italy	Cross-sectional	A	236	57.2	34.5	23.5
Varga et al. [95]	2014	Hungary	Cross-sectional	C	810	89.4	32.4	23.2
Voglino et al. [96]	2021	Italy	Cross-sectional	E	240	68.8	44.0	Not reported
Yeşildemir and Tek [97]	2022	Turkey	Cross-sectional	B	206	50.0	26.2	24.2
Yılmaz & Dundar [98]	2022	Turkey	Cross-sectional	C	248	41.9	42.6	25.3
Yilmazel [99]	2021	Turkey	Cross-sectional	A	969	63.9	21.4	Not reported
Yilmazel and Bozdogan [100]	2020	Turkey	Cross-sectional	C	420	46.2	43.4	Not reported
Zańko et al. [101]	2019	Poland	Cross-sectional	A	87	87.0	21.0	21.6

BMI – body mass index

*A – general population, B – people focused on sports performance or body composition, C – people from health-related programs or professions, D – people living with the disease, E – people with a special diet.

In this systematic review and meta-analysis, 34 studies used the <35 cutoff point [20,29,30,33,37,38,40,41,46,49,55,56,59,60,64-67,69-71,73-77,81-84,88,90,92,93,96], 64 studies used the <40 cutoff point [10,20,29-39,42-45,47-54,56-58,61-65,68,70,72-76,78-101], and 23 studies used both cutoff points [20,29,30,33,38,49,56,64,65,70,73-76,81-84,88,90,92,93,96]. Based on the <35 cutoff point, all the studies were cross-sectional. According to sex, 64 studies reported the overall proportion of ON symptoms in both men and women [10,29-38,40-44,46-50,53-55,57-59,61-68,71,73-91,93-101], and nine studies only included one sex (i.e. only women [39,45,51,52,60,70,72,92] or men [69]). The remaining two studies [20,56] did not report proportions stratified by sex. In terms of geographical regions, 18 different countries were identified, including 35 studies in Europe [20,32,33,35,38-43,46,55-60,65,66,70,74-79,81-83,88,90,94-96,101], 18 in Asia [29,31,34,37,47,48,51-53,61,62,68,87,89,97-100], 12 in South America [36,44,45,50,54,67,72,73,80,85,86,91], five in North America [10,49,63,71,93], and one in Oceania [84]. Two studies did not specify the country since it was performed via social media [64,92]. Two studies were conducted in two countries [30,69].

### Risk of study bias

Globally, using the modified version of the quality assessment tool by Hoy et al. [24], a higher risk of bias was observed in questions one and four, that is, those relating to the external validity of the studies. Individually, two studies presented a moderate risk of bias [34,78], presenting four points. The remaining 73 studies were deemed to be at low risk of bias, presenting scores between zero and three points. 19 studies presented three points [30,31,38-40,42,46,50-52,54,55,57,59,64,67,76,86,90]. The main sources of bias were related to the representativeness of the analysed sample [10,20,30-52,54-60,63-68,70-76,78-80,82,84-93,95-101]. A summary of the risk of bias scoring is shown in Table S5 in the **Online Supplementary Document**.

### Results of syntheses

#### Orthorexia nervosa symptoms

**Figure 2:**
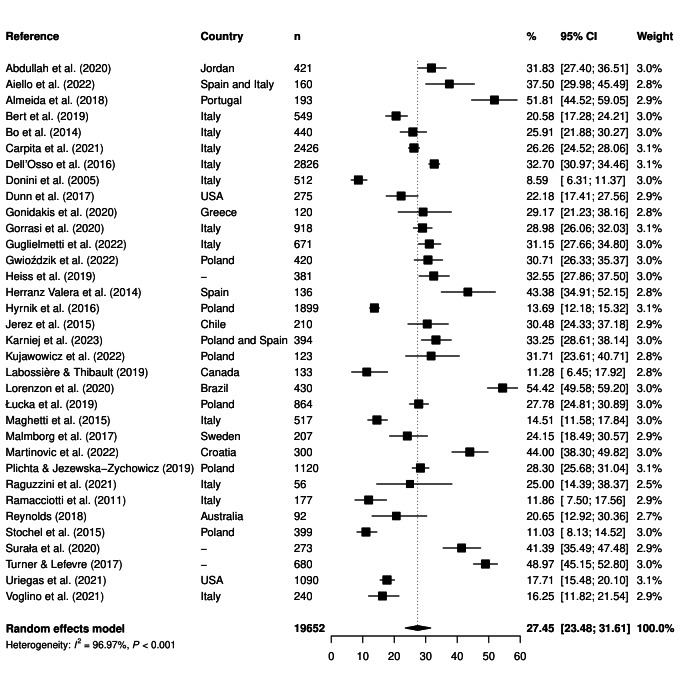
The overall proportion of orthorexia nervosa symptoms (using the cutoff <35 points). CI – confidence interval, *I^2^* – heterogeneity statistic, n – sample

**Figure 2** shows that the overall proportion of ON symptoms (using the cutoff <35 points) was 27.5% (95% CI = 23.5-31.6, *I^2^* = 97.0%, n = 34). The LFK index for the Doi plots showed no asymmetry of publication bias (LFK index = 0.08) (provided in Figure S1 in the **Online Supplementary Document**). The overall proportion of ON symptoms using the cutoff <40 points can be found in Figure S2 in the **Online Supplementary Document**. In addition, in Figure S3 in the **Online Supplementary Document**, further analysis according to the representativeness of the study samples can be found. No significant differences were observed according to the representativeness of the sample.

**Figure 3:**
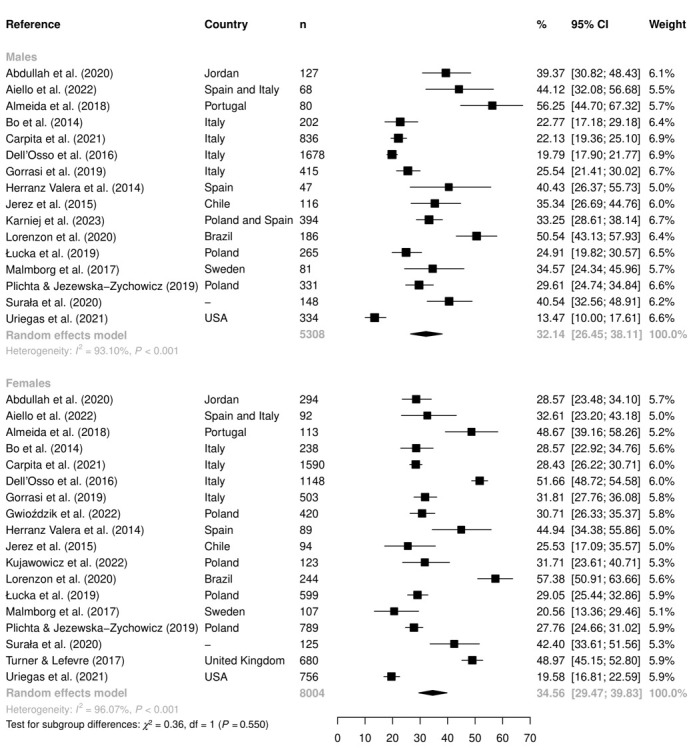
The overall proportion of orthorexia nervosa symptoms (using the cutoff <35 points) according to sex. *χ^2^* – Chi-squared test, CI – confidence interval, df – degrees of freedom, *I^2^* – heterogeneity statistic, n – sample

**Figure 3** shows the subgroup analysis in relation to sex. Using the cutoff <35 points, the overall proportion of ON symptoms for female sex was 34.6% (95% CI = 29.5-39.8, *I^2^* = 96.1%, n = 18), and for male sex, it was 32.1% (95% CI = 26.5-38.1, *I^2^* = 93.1%, n = 16), with no significant differences between sexes (*P* = 0.550).

**Figure 4:**
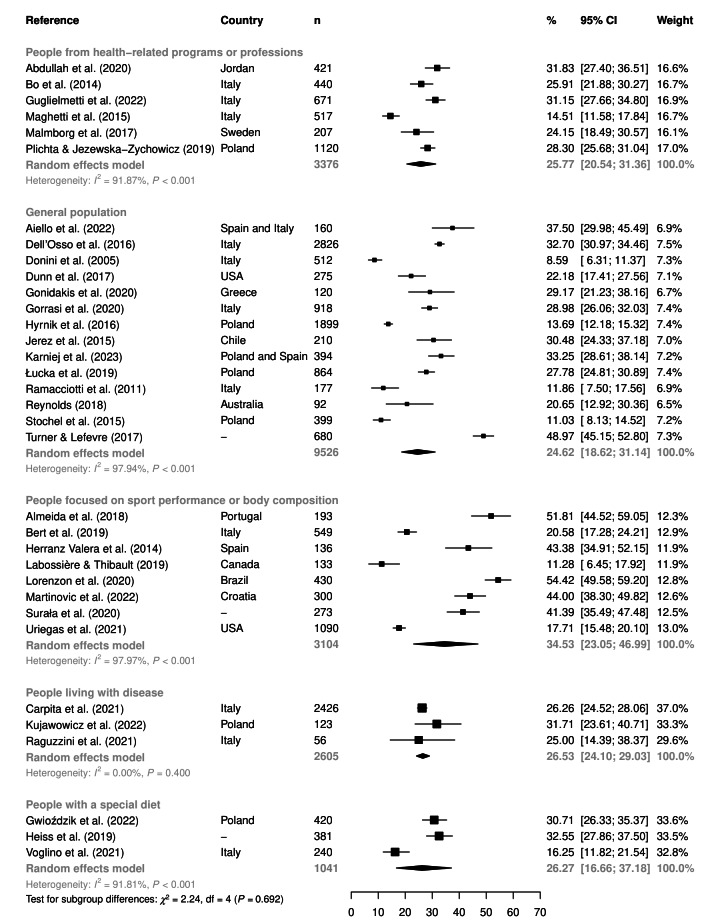
The overall proportion of orthorexia nervosa symptoms (using the cutoff <35 points) according to the type of population. *χ^2^* – Chi-squared test, CI – confidence interval, df – degrees of freedom, *I^2^* – heterogeneity statistic, n – sample

A subgroup analysis regarding the type of population (i.e. general population, people focused on sports performance or body composition, people from health-related programs or professions, people living with the disease, or people with a special diet) is shown in **Figure 4**. Using the cutoff <35 points, the highest overall proportion was found in people focused on sports performance or body composition (34.5%, 95% CI = 23.1-47.0, *I^2^* = 98.0%, n = 8). However, no significant differences were found in comparison with other types of populations (*P* = 0.692).

**Figure 5:**
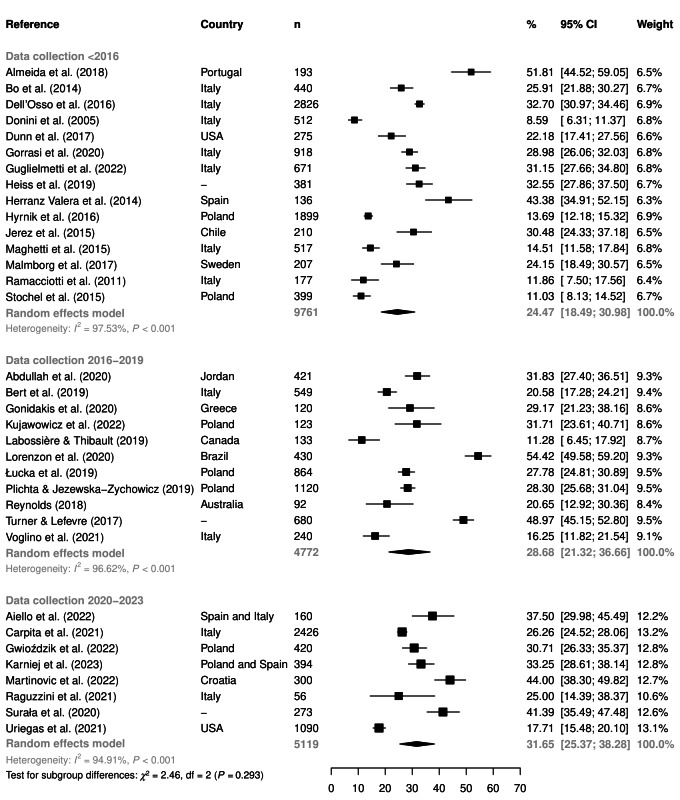
The overall proportion of orthorexia nervosa symptoms (using the cutoff <35 points) according to the period of data collection. *χ^2^* – Chi-squared test, CI – confidence interval, df – degrees of freedom, *I^2^* – heterogeneity statistic, n – sample

**Figure 5** shows another subgroup analysis according to the data collection year of the studies (i.e. prior to 2016, between 2016 and 2019, or between 2020 and 2023). Although no statistically significant differences were found (*P* = 0.293), a temporal increase in the proportions of ON symptoms over the years was observed, with the highest prevalence in the most recent studies (i.e. 2020 to 2023) (31.7%, 95% CI = 25.4-38.3, *I^2^* = 94.9%, n = 8).

The random-effects meta-regression models of body mass index or mean age with regard to the overall proportion of ON symptoms (using the cutoff <35 points) are shown in **Table 2**. The proportion of ON symptoms was not associated with either body mass index (unstandardised beta coefficient (*B*) = 0.007; 95% CI = -0.024 to 0.038, *P* = 0.666, n = 21) or mean age (*B* = 0.002; 95% CI = -0.003 to 0.007, *P* = 0.482, n = 30). Conversely, the data collection date was associated with a higher overall proportion of ON symptoms (*B* = 0.014; 95% CI = 0.005-0.024, *P* = 0.005, n = 34) (i.e. over the years, the proportion of ON symptoms was higher).

**Table 2:** Random-effects meta-regression models of body mass index and mean age of overall proportion of orthorexia nervosa symptoms

Variables	n of studies	*B*	SE	LLCI	ULCI	*P*-value
**ORTO-15** **(<35 cutoff point)**						
BMI (kilogramme/square metre)	21	0.007	0.016	-0.024	0.038	0.666
Mean age (years)	30	0.002	0.003	-0.003	0.007	0.482
Data collection date (years)	32	0.014	0.005	0.004	0.024	0.005

n – sample, *B* – unstandardised beta coefficient, BMI – body mass index, SE – standard error, LLCI – lower limit confidence interval, ULCI – upper limit confidence interval

## DISCUSSION

To our knowledge, this is the first meta-analysis that has comprehensively examined the overall proportion of ON symptoms, as well as in terms of sex, type of population, data collection date, body mass index, and mean age. Overall, our findings indicated that approximately three out of 10 study participants showed ON symptoms according to the ORTO-15 tool (using the cutoff <35 points). However, caution is required when interpreting this result, given that only 17.6% of the studies using this cutoff included representative samples. Although we did not observe significant differences in the ON symptom estimates based on the representativeness of the samples, further research could modify our confidence in the estimate [24]. Our findings are in line with previous systematic reviews that estimated the proportion of this type of eating disorder [14,15]. This high proportion could be explained in part by some of the following reasons. One of the main reasons could be related to the psychometric limitations of the ORTO-15 to reliably measure ON flaws [6,102]. This may also explain why proportion rates are much higher than those of other eating disorders [14,15]. Notwithstanding, it must be considered that there are no reliable studies reporting the prevalence of ON [3]. Furthermore, ON symptoms are related to restraint and weight loss efforts. It is possible that the ORTO-15 items consider people with ON symptoms to be those who are on a diet or control their food intake, which may lead to an overestimation of the overall proportion of ON symptoms [103]. In addition, some of the psychological and behavioural aspects of eating disorders are shared by people who are at risk of ON [104], which could overestimate the results found. Nevertheless, ON symptoms are not related to body dissatisfaction or dysregulated eating, which suggests that ON may represent a distinct eating disorder [103]. Another possible reason is that ON involves, in addition to a pathological dimension, a nonpathological interest in healthy eating, which has been called healthy ON [9]. However, until now, ON and healthy ON have been treated as essentially equivalent [105].

On the other hand, we did not observe significant differences in the proportion of ON symptoms between females and males. Scientific literature is inconsistent with regard to the relationship between sex and ON symptoms. A higher proportion of ON symptoms has been found in females than in men in several countries [6,46,87,104,106]. However, these samples were predominantly female (ranging from 58.0% to 74.6%), which may influence the results obtained [21]. Furthermore, of the 34 studies that used the ORTO-15 cutoff point of <35 points, 29 included participants of both sexes and five of only one sex (i.e. females only or males only). Of those studies, only 19 (65.5%) presented data separately for males and females. This fact added to the lack of representativeness in many studies as well as the heterogeneity of the population groups and the different contexts examined, and it is important to exercise caution when interpreting the findings from these studies.

Importantly, we observed a greater proportion of ON symptoms when studies assessed exclusively included people focused on sports performance or body composition reported the highest proportion of ON symptoms than in other populations (i.e. people from health-related programs or professions, the general population, people living with the disease, or people with a special diet). This is in line with a previous review by Hafstad et al. [22], which found that the overall prevalence of ON in exercising populations was very high. One possible explanation is that individuals who are concerned about controlling diet and calorie intake to improve their physical performance or body composition may be afraid of gaining body weight, which could lead to increased ON symptoms [107]. Another possible explanation could lie in the hypothesis that ON may be related to the personality profile and that those who are anxiety-prone and/or perfectionists may be more sensitive to developing these symptoms [108]. This hypothesis could explain the higher proportion of ON symptoms in certain populations (e.g. athletes, fitness practitioners) [98]. Notwithstanding, caution should be exercised in interpreting this result, as reverse causality could be a potential pitfall in this relationship. Therefore, further prospective studies are required to elucidate the direction of this association as well as the possible mechanisms involved in this association. In addition, very few studies have been conducted specifically on people from health-related programs/professions. Furthermore, it is possible that some of the studies focused on the general population may also include people from health-related programs or professions, without segmenting the data by this category.

Additionally, we observed an increasing trend over the years in relation to the proportion of ON symptoms in the studies found in the scientific literature. Although information on trends in these symptoms is scarce, there are some possible reasons explaining this result. For instance, the use of social networks could favour behaviours focused on perfectionism, dieting, poor body image and striving for thinness, which have been associated with increased ON symptoms [21] and could explain (at least in part) this finding. Social media can promote idealised images of “perfect” bodies and lifestyles, including highly restrictive eating [109]. Similarly, social media frequently showcase images of individuals who adhere to prevailing beauty standards [110], which could result in social comparison, potentially heightening feelings of dissatisfaction with one’s body and promoting unhealthy eating behaviours [111]. Supporting this notion, high social network use [112,113], and addictive behaviours [113] have also been associated with greater odds of presenting disordered eating (including ON [112]). More specifically, it has been suggested that the healthy eating community on Instagram (i.e. a social network) reports a high prevalence of ON symptoms, with higher use of this social network being associated with greater symptoms [114]. On the other hand, our results indicated that the period with the highest prevalence was after the coronavirus disease 2019 (COVID-19) lockdown. This result is in line with previous studies (using different tools than the ORTO-15 to assess ON symptoms) that observed increased symptoms during this period [115]. The increase in time spent on social media due to the COVID-19 pandemic has been identified as one of the reasons for the increase in ON symptoms [116]. In addition, the change in people’s diets due to the fear and stress caused by the COVID-19 pandemic seems to have led to an increased obsession with healthy eating [117].

Our results also showed that neither body mass index nor mean age was associated with the proportion of ON symptoms. Regarding body mass index, this result agrees with the scientific literature [21]. Studies analysing the relationship between body mass index and ON symptoms are inconsistent, with higher-quality studies showing a lack of association [6,87,104,106]. Likewise, ON symptoms are associated with the consumption of healthy and non-processed food with the aim of being healthy (and not losing weight as in the case of eating disorders) [118], which may explain (at least in part) this finding. In relation to mean age, we found no association with the overall proportion of ON symptoms. This finding agrees with the systematic review by McComb et al. [21], who indicated mixed findings regarding the role of age as a risk factor for ON. Although several studies have found a higher proportion of ON symptoms in younger adults than in older adults, there is mixed evidence with regard to the role of age as a risk factor for ON symptoms [46].

Although much of the scientific literature has relied on the ORTO-15 for assessing ON and is the most commonly used tool for assessing ON symptoms on a global scale, Atchison et al. [103] excluded data from the ORTO-15 in their systematic review, claiming its lack of validity and reliability, and because these authors considered that there is a lack of clarity on whether it measures healthy ON, ON, both, or neither. Other authors have also suggested that the use of the ORTO-15 questionnaire to diagnose ON is questionable due to a high percentage of false-positive results [14]. Despite these methodological limitations, our systematic review and meta-analysis contribute to scientific knowledge by quantifying, for the first time, the overall proportion of ON symptoms on a global scale based on the ORTO-15 tool, as well as identifying specific groups or moderator variables that could be associated with ON symptoms. This study is also in line with the World Health Organization’s Comprehensive mental health action plan 2013-2030, which has established certain priorities/goals, such as strengthening the evidence, information systems, and research for mental health (among others), because of the crucial role that mental health plays in people’s well-being [119].

The current systematic review and meta-analysis has several limitations that must be recognised. We included the overall proportion of ON symptoms assessed by the ORTO-15, which has several methodological flaws [6,102,106]. However, none of the tools have been used as “a gold standard” (i.e. the most suitable tool for ON evaluation). Because of the substantial diagnostic differences between the existing tools, a new concept of diagnostic criteria is needed, and consequently, a new tool for screening these disorders must be developed [120]. Future studies should make use of tools with more adequate psychometric properties when assessing the prevalence/proportion of ON symptoms [102] (i.e. Teruel Orthorexia Scale [9]). Given that the ORTO-15 and its modified versions continue to be widely used in studies examining ON, it is imperative to reiterate previous suggestions made by some authors against using this tool or any of its alternative forms for ON assessment [6,102,106]. Furthermore, the studies included in the present article used self-report questionnaires to determine ON symptoms, which may lead to both recall bias and social desirability. In addition, grey literature was not included in this review. This could have led to losses of information for the results obtained. Moreover, a great number of studies did not include representative samples of participants. This fact, coupled with this being a popular and trendy topic, may introduce a bias and potentially lead to inflated estimates of ON symptoms. In addition, most of the data came from European countries, while there were no studies performed in African countries. Thus, future studies should also ensure the inclusion of representative samples in different countries (especially in those not studied or little studied) to establish a more accurate picture of the overall situation of ON symptoms. Therefore, the results cannot be generalised to the global population and should be interpreted with caution. Despite these limitations, these results can inform intervention priorities for preventing ON symptoms as a global health initiative to avoid health-related problems among the general population [119], particularly in individuals focused on improving their physical performance and/or body composition. Additionally, it would be interesting for future studies to not only include cases of participants with ON symptoms at the global level but also stratify by numerous factors, such as sex, type of population, or body mass index status. Additionally, given the high prevalence of disordered eating in adolescents [121], that ON is a growing health concern, that the proportion of ON symptoms seems to be increasing over the years and that some studies include samples within that age range (when the ORTO-15 tool has not been validated for this population), validation of these tools in the adolescent population is strongly recommended.

## CONCLUSIONS

To conclude, the available evidence indicates that approximately three out of 10 study participants showed ON symptoms according to the ORTO-15 tool. There were no substantial differences in the proportion of ON symptoms by sex. These high percentages and the increasing temporal trend are worrisome from a public health perspective and highlight the need to develop psychometric instruments to aid in clinical diagnosis and treatment efficacy [3]. Furthermore, it is urged to prioritise the utilisation of alternative measures to the ORTO-15 questionnaire that have been developed in the past decade to screen for ON symptoms [102].

## Additional material


Online Supplementary Document
