# Supplementary material for: Overall proportion of orthorexia nervosa symptoms: A systematic review and meta-analysis including 30 476 individuals from 18 countries
Source: J Glob Health. 2023 Nov 3;13:04087. doi: 10.7189/jogh.13.04087 (PMC10622118; doi:10.7189/jogh.13.04087)
Supplement: Online Supplementary Document [file jogh-13-04087-s001.pdf]

Figure S1. Doi plots for Luis Furuya-Kanamori index. LFK – Luis Furuya-Kanamori, ON – orthorexia nervosa.

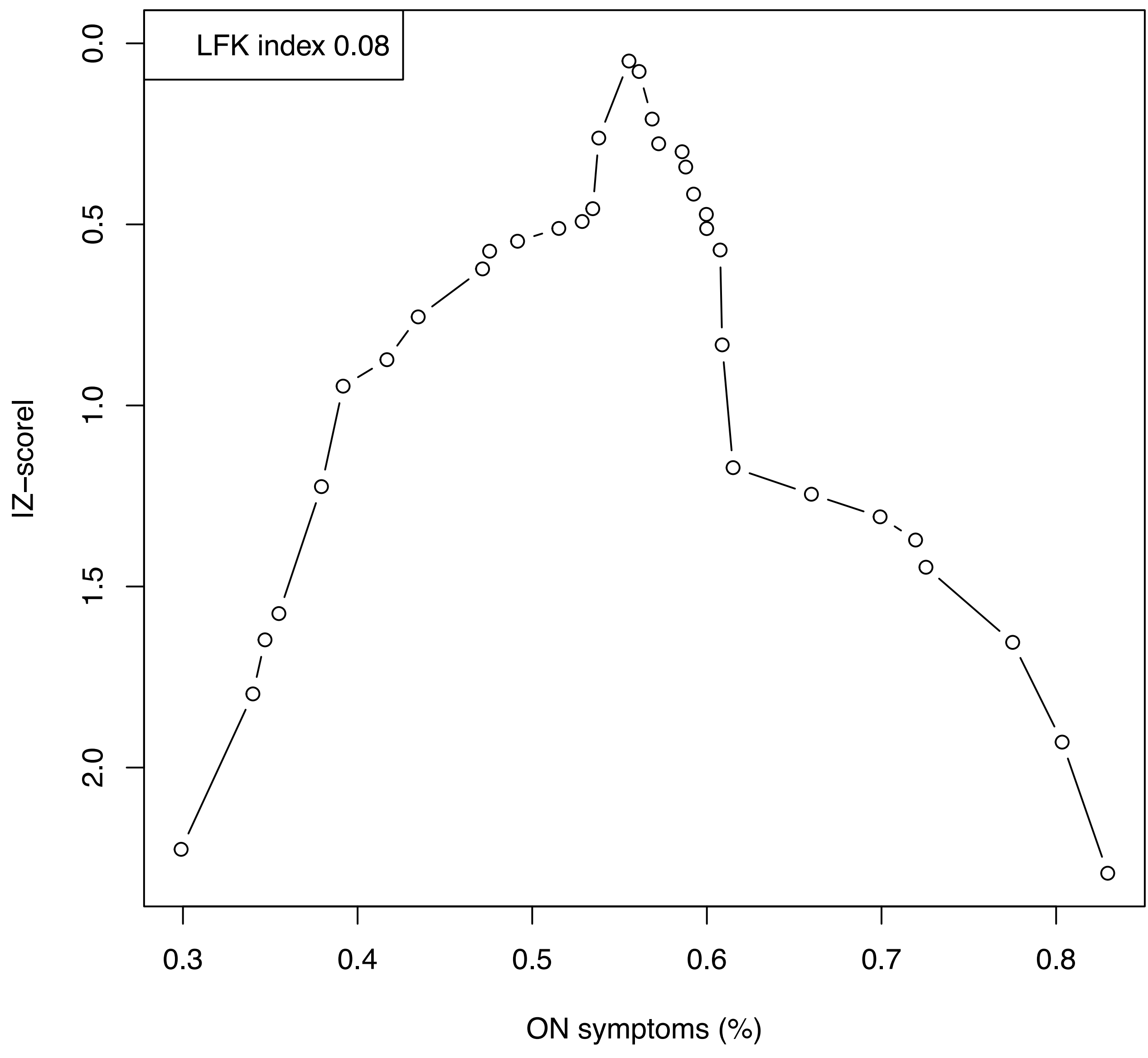

Figure S2. Overall proportion of orthorexia nervosa symptoms (using the cut-off <40 points). CI – confidence interval, n – sample, I^2 – heterogeneity statistic, P – P value.

Reference	Country	n	%	95% CI	Weight
Abdullah et al. (2020)	Jordan	421	71.97	[67.42; 76.21]	1.6%
Aiello et al. (2022)	Spain and Italy	160	82.50	[75.71; 88.05]	1.6%
Aksoydan & Camci (2009)	Turkey	94	56.38	[45.76; 66.59]	1.5%
Albery et al. (2019)	United Kingdom	80	30.00	[20.26; 41.28]	1.5%
Almeida et al. (2018)	Portugal	193	89.12	[83.85; 93.14]	1.6%
Asil & Sürücüoğlu (2015)	Turkey	117	41.88	[32.82; 51.36]	1.5%
Athanasaki et al. (2023)	Greece	96	73.96	[64.00; 82.38]	1.5%
Bergonzi & Massarollo (2022)	Brazil	61	44.26	[31.55; 57.55]	1.5%
Bert et al. (2019)	Italy	549	69.22	[65.17; 73.06]	1.6%
Bién & Pieczykolan (2017)	Poland	280	71.43	[65.75; 76.65]	1.6%
Bosi et al. (2007)	Turkey	315	45.40	[39.81; 51.07]	1.6%
Clifford & Blyth (2019)	United Kingdom	215	40.93	[34.29; 47.82]	1.6%
Cosentino et al. (2023)	Italy	88	69.32	[58.58; 78.71]	1.5%
De Marchi & Baratto (2018)	Brazil	82	80.49	[70.26; 88.42]	1.5%
de Souza & Rodrigues (2014)	Brazil	150	88.67	[82.48; 93.26]	1.6%
Demir & Bayram (2022)	Turkey	310	28.71	[23.74; 34.09]	1.6%
Demirer & Yardımcı (2023)	Turkey	197	71.07	[64.19; 77.29]	1.6%
Donini et al. (2005)	Italy	512	30.08	[26.13; 34.26]	1.6%
Dunn et al. (2016)	USA	275	71.27	[65.53; 76.55]	1.6%
Elias et al. (2022)	Brazil	246	72.76	[66.74; 78.23]	1.6%
Erdogan et al. (2022)	Turkey	159	75.47	[68.03; 81.94]	1.6%
Ermumcu & Tek (2018)	Turkey	132	75.76	[67.53; 82.79]	1.6%
Farchakh et al. (2019)	Lebanon	627	74.48	[70.88; 77.85]	1.6%
Freire et al. (2020)	Brazil	60	80.00	[67.67; 89.22]	1.5%
Gonidakis et al. (2020)	Greece	120	55.00	[45.65; 64.09]	1.5%
Grajek et al. (2022)	Poland	290	44.48	[38.67; 50.41]	1.6%
Gubiec et al. (2015)	Poland	155	58.71	[50.53; 66.55]	1.6%
Haddad et al. (2019)	Lebanon	806	73.08	[69.87; 76.11]	1.6%
Hamid et al. (2018)	Malaysia	138	67.39	[58.90; 75.12]	1.6%
Hayles et al. (2017)	USA	404	79.70	[75.45; 83.52]	1.6%
Heiss et al. (2019)	–	381	77.69	[73.17; 81.77]	1.6%
Herranz Valera et al. (2014)	Spain	136	86.03	[79.05; 91.37]	1.6%
Karadağ et al. (2016)	Turkey	750	50.40	[46.76; 54.04]	1.6%
Koven & Senbonmatsu (2013)	USA	42	50.00	[34.19; 65.81]	1.4%
Kujawowicz et al. (2022)	Poland	123	70.73	[61.85; 78.59]	1.6%
Lemos et al. (2018)	Brazil	95	70.53	[60.29; 79.44]	1.5%
Lorenzon et al. (2020)	Brazil	430	91.40	[88.33; 93.87]	1.6%
Łucka et al. (2019)	Poland	864	76.74	[73.77; 79.52]	1.6%
Maghetti et al. (2015)	Italy	517	54.93	[50.53; 59.28]	1.6%
Malmborg et al. (2017)	Sweden	207	69.57	[62.81; 75.75]	1.6%
Mitrofanova et al. (2021)[a]	Greece	10	80.00	[44.39; 97.48]	1.0%
Mitrofanova et al. (2021)[b]	United Kingdom	50	64.00	[49.19; 77.08]	1.4%
Penaforte et al. (2017)	Brazil	141	87.23	[80.58; 92.26]	1.6%
Plichta & Jezewska–Zychowicz (2019)	Poland	1120	75.00	[72.36; 77.51]	1.6%
Raguzzini et al. (2021)	Italy	56	64.29	[50.36; 76.64]	1.5%
Ramacciotti et al. (2011)	Italy	177	57.63	[49.99; 65.01]	1.6%
Reynolds (2018)	Australia	92	66.30	[55.70; 75.83]	1.5%
Rizzieri et al. (2019)	Brazil	65	89.23	[79.06; 95.56]	1.5%
Sampaio et al. (2022)	Brazil	150	80.00	[72.70; 86.08]	1.6%
Sanlier et al. (2016)	Turkey	900	59.78	[56.49; 63.00]	1.6%
Stochel et al. (2015)	Poland	399	53.13	[48.10; 58.12]	1.6%
Sünbül & Bayrak (2021)	Turkey	580	39.83	[35.82; 43.94]	1.6%
Surała et al. (2020)	–	273	88.28	[83.86; 91.84]	1.6%
Tocchetto et al. (2018)	Brazil	50	78.00	[64.04; 88.47]	1.4%
Turner & Lefevre (2017)	United Kingdom	680	90.59	[88.14; 92.68]	1.6%
Uriegas et al. (2021)	USA	1090	67.98	[65.12; 70.75]	1.6%
Vaccari et al. (2021)	Italy	236	58.90	[52.33; 65.24]	1.6%
Varga et al. (2014)	Hungary	810	74.20	[71.04; 77.18]	1.6%
Voglino et al. (2021)	Italy	240	61.25	[54.77; 67.45]	1.6%
Yeşildemir & Tek (2022)	Turkey	206	81.55	[75.57; 86.60]	1.6%
Yilmazel (2021)	Turkey	969	64.40	[61.29; 67.42]	1.6%
Yilmazel & Bozdogan (2020)	Turkey	420	78.57	[74.33; 82.40]	1.6%
Yılmaz & Dundar (2022)	Turkey	248	18.95	[14.27; 24.39]	1.6%
Zańko et al. (2019)	Poland	87	41.38	[30.92; 52.45]	1.5%
**Random effects model**		**19926**	**66.96**	**[62.57; 71.20]**	**100.0%**

Heterogeneity: $I^2 = 97.38\%$, $P < 0.001$

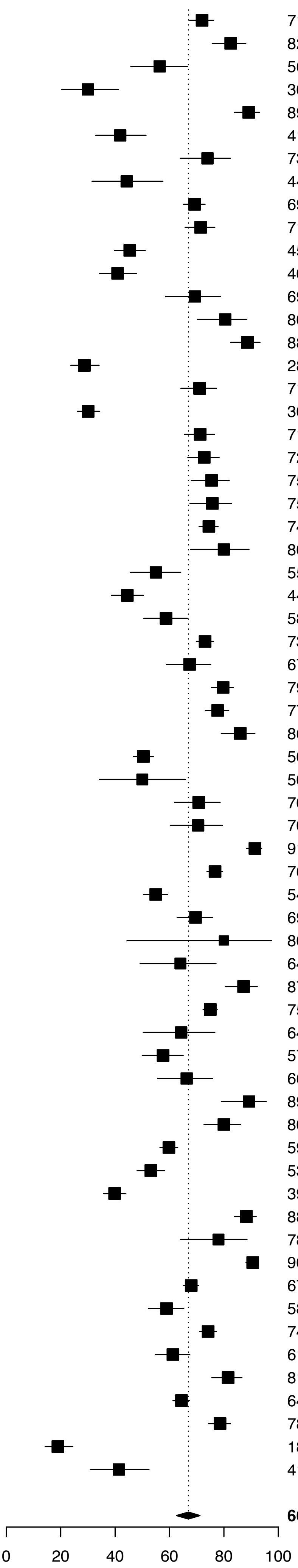

Figure S3. Overall proportion of orthorexia nervosa symptoms (using the cut-off <35 points) according to the representativeness of the sample. χ^2 – chi-squared test, CI – confidence interval, df – degrees of freedom, n – sample, I^2 – heterogeneity statistic, *P* – P value.

Reference	Country	n	%	95% CI	Weight
**Nonrepresentative samples**					
Aiello et al. (2022)	Spain and Italy	160	37.50	[29.98; 45.49]	3.5%
Almeida et al. (2018)	Portugal	193	51.81	[44.52; 59.05]	3.5%
Bert et al. (2019)	Italy	549	20.58	[17.28; 24.21]	3.7%
Bo et al. (2014)	Italy	440	25.91	[21.88; 30.27]	3.7%
Carpita et al. (2021)	Italy	2426	26.26	[24.52; 28.06]	3.8%
Dell’Osso et al. (2016)	Italy	2826	32.70	[30.97; 34.46]	3.8%
Donini et al. (2005)	Italy	512	8.59	[6.31; 11.37]	3.7%
Dunn et al. (2017)	USA	275	22.18	[17.41; 27.56]	3.6%
Gonidakis et al. (2020)	Greece	120	29.17	[21.23; 38.16]	3.4%
Gorrasi et al. (2020)	Italy	918	28.98	[26.06; 32.03]	3.7%
Guglielmetti et al. (2022)	Italy	671	31.15	[27.66; 34.80]	3.7%
Gwioździk et al. (2022)	Poland	420	30.71	[26.33; 35.37]	3.6%
Heiss et al. (2019)	–	381	32.55	[27.86; 37.50]	3.6%
Herranz Valera et al. (2014)	Spain	136	43.38	[34.91; 52.15]	3.4%
Hyrnik et al. (2016)	Poland	1899	13.69	[12.18; 15.32]	3.7%
Jerez et al. (2015)	Chile	210	30.48	[24.33; 37.18]	3.5%
Kujawowicz et al. (2022)	Poland	123	31.71	[23.61; 40.71]	3.4%
Labossière & Thibault (2019)	Canada	133	11.28	[6.45; 17.92]	3.4%
Lorenzon et al. (2020)	Brazil	430	54.42	[49.58; 59.20]	3.7%
Łucka et al. (2019)	Poland	864	27.78	[24.81; 30.89]	3.7%
Malmborg et al. (2017)	Sweden	207	24.15	[18.49; 30.57]	3.5%
Raguzzini et al. (2021)	Italy	56	25.00	[14.39; 38.37]	3.0%
Reynolds (2018)	Australia	92	20.65	[12.92; 30.36]	3.3%
Stochel et al. (2015)	Poland	399	11.03	[8.13; 14.52]	3.6%
Surała et al. (2020)	–	273	41.39	[35.49; 47.48]	3.6%
Turner & Lefevre (2017)	–	680	48.97	[45.15; 52.80]	3.7%
Uriegas et al. (2021)	USA	1090	17.71	[15.48; 20.10]	3.7%
Voglino et al. (2021)	Italy	240	16.25	[11.82; 21.54]	3.6%
**Random effects model**		**16723**	**27.65**	**[23.23; 32.30]**	**100.0%**
Heterogeneity: $I^2 = 97.19\%$, $P < 0.001$					
**Representative samples**					
Abdullah et al. (2020)	Jordan	421	31.83	[27.40; 36.51]	16.8%
Karniej et al. (2023)	Poland and Spain	394	33.25	[28.61; 38.14]	16.7%
Maghetti et al. (2015)	Italy	517	14.51	[11.58; 17.84]	16.9%
Martinovic et al. (2022)	Croatia	300	44.00	[38.30; 49.82]	16.5%
Plichta & Jezewska–Zychowicz (2019)	Poland	1120	28.30	[25.68; 31.04]	17.1%
Ramacciotti et al. (2011)	Italy	177	11.86	[7.50; 17.56]	16.0%
**Random effects model**		**2929**	**26.56**	**[17.30; 36.99]**	**100.0%**
Heterogeneity: $I^2 = 96.07\%$, $P < 0.001$					

Test for subgroup differences: $\chi^2 = 0.04$, df = 1 ($P = 0.841$)

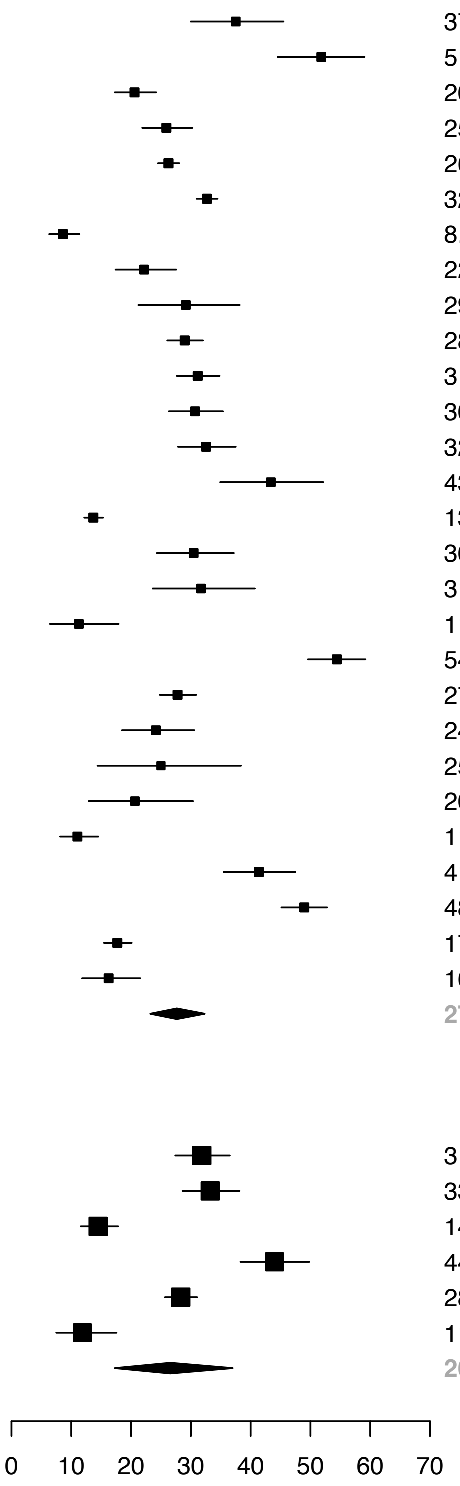

Table S1. Search strategy.

Databases searched until 26th June 2023.
Strategy for Web of Science:<br>Final search: TS=((Orthorexia OR "Orthorexia nervosa" OR "Orthorexic behaviors") AND ORTO-15) (No limitations were used).
**Results: 129**
Strategy for PubMed: ("Orthorexia nervosa"[MeSH Terms] OR ("orthorexia"[All Fields] AND "nervosa"[All Fields]) OR "Orthorexia nervosa"[All Fields] OR "orthorexia"[All Fields] OR "Orthorexia nervosa"[All Fields] OR "Orthorexic behaviors"[All Fields]) AND "ORTO-15"[All Fields]
**Results: 107**
Strategy for SCOPUS: (orthorexia nervosa OR orthorexic behaviour OR orthorexia) AND (orto-15) (No limitations were used).
**Results: 366**
Strategy for Cochrane: (Orthorexia):ti,ab,kw OR (Orthorexia nervosa):ti,ab,kw OR (Orthorexic behaviors):ti,ab,kw AND (ORTO-15):ti,ab,kw
**Results: 8**

Table 2. Excluded studies with reasons.

Aktürk, Ü., Gül, E., & Erci, B. (2019). The Effect of Orthorexia Nervosa Levels of Nursing Students and Diet Behaviors and Socio-Demographic Characteristics. Ecology of Food and Nutrition, 58(4), 397–409. https://doi.org/10.1080/03670244.2019.1602529
Reason for exclusion: ORTO cut-off point different than < 40 or < 35.
Alvarenga, M. S., Martins, M. C. T., Sato, K. S. C. J., Vargas, S. V. A., Philippi, S. T., & Scagliusi, F. B. (2012). Orthorexia nervosa behavior in a sample of Brazilian dietitians assessed by the Portuguese version of ORTO-15. Eating and Weight Disorders: EWD, 17(1), e29-35. https://doi.org/10.1007/BF03325325
Reason for exclusion: Proportion of disordered eating not shown.
Babeau, C., Le Chevanton, T., Julien-Sweerts, S., Brochenin, A., Donini, L. M., & Fouques, D. (2020). Structural validation of the ORTO-12-FR questionnaire among a French sample as a first attempt to assess orthorexia nervosa in France. Eating and Weight Disorders: EWD, 25(6), 1771–1778. https://doi.org/10.1007/s40519-019-00835-0
Reason for exclusion: Proportion of disordered eating not shown.
Barnes, M. A., & Caltabiano, M. L. (2017). The interrelationship between orthorexia nervosa, perfectionism, body image and attachment style. Eating and Weight Disorders: EWD, 22(1), 177–184. https://doi.org/10.1007/s40519-016-0280-x
Reason for exclusion: Proportion of disordered eating not shown.
Barnett, M. J., Dripps, W. R., & Blomquist, K. K. (2016). Organivore or organorexic? Examining the relationship between alternative food network engagement, disordered eating, and special diets. Appetite, 105, 713–720. https://doi.org/10.1016/j.appet.2016.07.008
Reason for exclusion: Proportion of disordered eating not shown.
Barrada, J. R., & Roncero, M. (2018). Bidimensional Structure of the Orthorexia: Development and Initial Validation of a New Instrument. Anales de Psicología, 34(2), 282–290. https://doi.org/10.6018/analesps.34.2.299671
Reason for exclusion: Proportion of disordered eating not shown.
Brytek-Matera, A., Plasonja, N., & Décamps, G. (2020). Assessing Orthorexia Nervosa: Validation of the Polish Version of the Eating Habits Questionnaire in a General Population Sample. Nutrients, 12(12). https://doi.org/10.3390/nu12123820
Reason for exclusion: Proportion of disordered eating not shown.
Brytek-Matera, A. (2020). Interaction between Vegetarian Versus Omnivorous Diet and Unhealthy Eating Patterns (Orthorexia Nervosa, Cognitive Restraint) and Body Mass Index in Adults. Nutrients, 12(3), 646. https://doi.org/10.3390/nu12030646
Reason for exclusion: No ORTO-15 used.
Brytek-Matera, A., Donini, L. M., Krupa, M., Poggiogalle, E., & Hay, P. (2015). Orthorexia nervosa and self-attitudinal aspects of body image in female and male university students. Journal of Eating Disorders, 3, 2. https://doi.org/10.1186/s40337-015-0038-2
Reason for exclusion: ORTO cut-off point different than < 40 or < 35.
Brytek-Matera, A., Fonte, M. L., Poggiogalle, E., Donini, L. M., & Cena, H. (2017). Orthorexia nervosa: Relationship with obsessive-compulsive symptoms, disordered eating patterns and body uneasiness among Italian university students. Eating and Weight Disorders: EWD, 22(4), 609–617. https://doi.org/10.1007/s40519-017-0427-4
Reason for exclusion: Proportion of disordered eating not shown.
Brytek-Matera, A., Krupa, M., Poggiogalle, E., & Donini, L. M. (2014). Adaptation of the ORTHO-15 test to Polish women and men. Eating and Weight Disorders: EWD, 19(1), 69–76. https://doi.org/10.1007/s40519-014-0100-0
Reason for exclusion: Proportion of disordered eating not shown.

Brytek-Matera, A., Pardini, S., Szubert, J., & Novara, C. (2022). Orthorexia Nervosa and Disordered Eating Attitudes, Self-Esteem and Physical Activity among Young Adults. Nutrients, 14(6), 1289. https://doi.org/10.3390/nu14061289
Reason for exclusion: No ORTO-15 used.
Brytek-Matera, A., Sacre, H., Staniszewska, A., & Hallit, S. (2020). The Prevalence of Orthorexia Nervosa in Polish and Lebanese Adults and Its Relationship with Sociodemographic Variables and BMI Ranges: A Cross-Cultural Perspective. Nutrients, 12(12), 3865. https://doi.org/10.3390/nu12123865
Reason for exclusion: No ORTO-15 used.
Brytek-Matera, A., Staniszewska, A., & Hallit, S. (2020). Identifying the Profile of Orthorexic Behavior and "Normal" Eating Behavior with Cluster Analysis: A Cross-Sectional Study among Polish Adults. Nutrients, 12(11), 3490. https://doi.org/10.3390/nu12113490
Reason for exclusion: No ORTO-15 used.
Brytek-Matera A, Obeid S, Donini LM, Rogoza M, Marchlewska M, Plichta M, et al. Psychometric properties of the ORTO-R in a community-based sample of women and men from Poland. Journal of Eating Disorders. 2023 Jan 19;11(1):9
Reason for exclusion: No ORTO-15 used.
Brytek-Matera, A., Obeid, S., Donini, L. M., Rogoza, M., Marchlewska, M., Plichta, M., Jezewska-Zychowicz, M., Hallit, S., & Rogoza, R. (2023). Psychometric properties of the ORTO-R in a community-based sample of women and men from Poland. Journal of Eating Disorders, 11(1), Art. 1. https://doi.org/10.1186/s40337-023-00734-x
Reason for exclusion: No ORTO-15 used.
Busatta, D., Cassioli, E., Rossi, E., Campanino, C., Ricca, V., & Rotella, F. (2022). Orthorexia among patients with eating disorders, student dietitians and general population: A pilot study. Eating and Weight Disorders: EWD, 27(2), 847–851. https://doi.org/10.1007/s40519-021-01184-7
Reason for exclusion: Proportion of disordered eating not shown.
Carlucci, L., Cinosi, E., Lupi, M., Matarazzo, I., Acciavatti, T., Sarchione, F., Santacroce, R., Martinotti, G., Giannantonio, M., D'Ambrosio, I., & Balsamo, M. (2016). L'ossessione del mangiare sano: La misurazione dell'«ortoressia nervosa» attraverso la scala Ortho-15. Psicoterapia Cognitiva e Comportamentale, 22, 367–372.
Reason for exclusion: Excluded due to data unavailability.
Cena, H., Porri, D., De Giuseppe, R., Kalmpourtzidou, A., Salvatore, F. P., El Ghoch, M., Itani, L., Kreidieh, D., Brytek-Matera, A., Pocol, C. B., Arteta Arteta, D. S., Utan, G., & Kolčić, I. (2021). How Healthy Are Health-Related Behaviors in University Students: The HOLISTic Study. Nutrients, 13(2), Article 2. https://doi.org/10.3390/nu13020675
Reason for exclusion: Proportion of disordered eating not shown.
Cerolini, S., Vacca, M., Zagaria, A., Donini, L. M., Barbaranelli, C., & Lombardo, C. (2022). Italian adaptation of the Düsseldorf Orthorexia Scale (I-DOS): Psychometric properties and prevalence of orthorexia nervosa among an Italian sample. Eating and Weight Disorders, 27(4), 1405-1413. https://doi.org/10.1007/s40519-021-01278-2
Reason for exclusion: Proportion of disordered eating not shown.
Dąbal, A. (2020). Characteristics and nosological separation of orthorexia nervosa. Psychiatria i Psychologia Kliniczna, 20, 32–42. https://doi.org/10.15557/PiPK.2020.0004
Reason for exclusion: ORTO cut-off point different than < 40 or < 35.
Dalga, D., & Yilmaz, H. O. (2021). The effect of nutrition education given to healthcare professionals on orthorexia nervosa. Progress in Nutrition, 23(2). https://doi.org/10.23751/pn.v23i2.10517
Reason for exclusion: ORTO cut-off point different than < 40 or < 35.

Dell'Osso, L., Carpita, B., Muti, D., Cremone, I. M., Massimetti, G., Diadema, E., Gesi, C., & Carmassi, C. (2018). Prevalence and characteristics of orthorexia nervosa in a sample of university students in Italy. Eating and Weight Disorders : EWD, 23(1), 55-65. https://doi.org/10.1007/s40519-017-0460-3
Reason for exclusion: Duplicated data.
Demir, H. P., & Bayram, H. M. (2022). Orthorexia nervosa: The relationship with obsessive-compulsive symptoms and eating attitudes among individuals with and without healthcare professionals. Mediterranean Journal of Nutrition and Metabolism, 15(1), 23–33. https://doi.org/10.3233/MNM-210015
Reason for exclusion: Excluded due to data unavailability.
Duradoni, M., Gursesli, M. C., Fiorenza, M., & Guazzini, A. (2023). The Relationship between Orthorexia Nervosa and Obsessive Compulsive Disorder. European Journal of Investigation in Health, Psychology and Education, 13(5), 861-869. https://doi.org/10.3390/ejihpe13050065
Reason for exclusion: No ORTO-15 used.
Duran, S., Çiçekoğlu, P., & Kaya, E. (2020). Relationship between orthorexia nervosa, muscle dysmorphic disorder (bigorexia), and self-confidence levels in male students. Perspectives in Psychiatric Care, 56(4), 878–884. https://doi.org/10.1111/ppc.12505
Reason for exclusion: ORTO cut-off point different than < 40 or < 35.
Duran, S., & Öz, Y. C. (2022). The relationships between Orthorexia nervosa, social appearance anxiety and women's self-esteem: A cross-sectional study. Bangladesh Journal of Medical Science, 21(3), Article 3. https://doi.org/10.3329/bjms.v21i3.59584
Reason for exclusion: ORTO cut-off point different than < 40 or < 35.
Elhabashy, S. A., Abd ElMalak, M. W., Elrassas, H. H., & Thabet, R. A. (2022). Disordered eating and behaviors among young Egyptians with type 1 diabetes: Risk factors and comorbidities. Journal of Pediatric Endocrinology & Metabolism: JPEM, 35(11), 1385–1393. https://doi.org/10.1515/jpem-2022-0336
Reason for exclusion: Excluded due to data unavailability.
Gerontidis, A., Grammatikopoulou, M. G., Tzimos, C., Gkiouras, K., Taousani, E., Athanasiadis, L., & Goulis, D. G. (2022). Effectors of Pregorexia and Emesis among Pregnant Women: A Pilot Study. Nutrients, 14(24), Article 24. https://doi.org/10.3390/nu14245275
Reason for exclusion: Proportion of disordered eating not shown.
Gkiouras, K., Grammatikopoulou, M. G., Tsaliki, T., Ntwali, L., Nigdelis, M. P., Gerontidis, A., Taousani, E., Tzimos, C., Rogoza, R., Bogdanos, D. P., Donini, L. M., & Goulis, D. G. (2022). Orthorexia nervosa: Replication and validation of the ORTO questionnaires translated into Greek in a survey of 848 Greek individuals. Hormones (Athens, Greece), 21(2), 251–260. https://doi.org/10.1007/s42000-022-00351-4
Reason for exclusion: Proportion of disordered eating not shown.
Gorrasi, I. S. R., Ferraris, C., Degan, R., Daga, G. A., Bo, S., Tagliabue, A., Guglielmetti, M., Roppolo, M., Gilli, G., Maran, D. A., & Carraro, E. (2022). Use of online and paper-and-pencil questionnaires to assess the distribution of orthorexia nervosa, muscle dysmorphia and eating disorders among university students: Can different approaches lead to different results? Eating and Weight Disorders: EWD, 27(3), 989–999. https://doi.org/10.1007/s40519-021-01231-3
Reason for exclusion: Proportion of disordered eating not shown.
Grajek, M., Kobza, J., Sobczyk, K., Działach, E., Białek-Dratwa, A., Górski, M., & Sas-Nowosielski, K. (2020). Dietary habits and orthorexic behaviours of dancing school female students. Journal of Physical Education and Sport, 3102–3107. https://doi.org/10.7752/jpes.2020.s6421
Reason for exclusion: ORTO cut-off point different than < 40 or < 35.
Gramaglia, C., Brytek-Matera, A., Rogoza, R., & Zeppegno, P. (2017). Orthorexia and anorexia nervosa: Two distinct phenomena? A cross-cultural comparison of orthorexic behaviours in clinical and non-clinical samples. BMC Psychiatry, 17(1), 75. https://doi.org/10.1186/s12888-017-1241-2

Reason for exclusion: Subjects with eating disorder.
Gramaglia, C., Gambaro, E., Delicato, C., Marchetti, M., Sarchiapone, M., Ferrante, D., Roncero, M., Perpiñá, C., Brytek-Matera, A., Wojtyna, E., & Zeppegno, P. (2019). Orthorexia nervosa, eating patterns and personality traits: A cross-cultural comparison of Italian, Polish and Spanish university students. BMC Psychiatry, 19(1), 235. https://doi.org/10.1186/s12888-019-2208-2
Reason for exclusion: Proportion of disordered eating not shown.
Grammatikopoulou, M. G., Gkiouras, K., Markaki, A., Theodoridis, X., Tsakiri, V., Mavridis, P., Dardavessis, T., & Chourdakis, M. (2018). Food addiction, orthorexia, and food-related stress among dietetics students. Eating and Weight Disorders: EWD, 23(4), 459–467. https://doi.org/10.1007/s40519-018-0514-1
Reason for exclusion: No ORTO-15 used.
Haddad, C., Hallit, R., Akel, M., Honein, K., Akiki, M., Kheir, N., Obeid, S., & Hallit, S. (2020). Validation of the Arabic version of the ORTO-15 questionnaire in a sample of the Lebanese population. Eating and Weight Disorders: EWD, 25(4), 951–960. https://doi.org/10.1007/s40519-019-00710-y
Reason for exclusion: Proportion of disordered eating not shown.
Hyrnik, J., Janas-Kozik, M., Stochel, M., Jelonek, I., Siwiec, A., Krysta, K., & Rybakowski, J. K. (2016). Prevalence of orthorexia nervosa among polish adolescents – Assessment made by the ORTO-15 Questionnaire. European Psychiatry, 33, S430. https://doi.org/10.1016/j.eurpsy.2016.01.1555
Reason for exclusion: Duplicated data.
James, C., Harrison, A., Seixas, A., Powell, M., Pengpid, S., & Peltzer, K. (2017). “Safe Foods” or “Fear Foods”: The implications of food avoidance in college students from low- and middle-income countries. Eating and Weight Disorders: EWD, 22(3), 407–419. https://doi.org/10.1007/s40519-017-0407-8
Reason for exclusion: No ORTO-15 used.
Kalika, E., Egan, H., & Mantzios, M. (2022). Exploring the role of mindful eating and self-compassion on eating behaviours and orthorexia in people following a vegan diet. Eating and Weight Disorders: EWD, 27(7), 2641–2651. https://doi.org/10.1007/s40519-022-01407-5
Reason for exclusion: No ORTO-15 used.
Kamarli Altun, H., Keser, I., & Bozkurt, S. (2020). Comparison of Eating Attitudes and the Susceptibility to Orthorexia Nervosa of Students in Health-Related Fields and Those in Other Fields. Iranian Journal of Public Health. https://ijph.tums.ac.ir/index.php/ijph/article/view/19878
Reason for exclusion: ORTO cut-off point different than < 40 or < 35.
Kaźmierczak-Wojtaś, N., & Drozd, M. (2022). Diet Quality and Level of Nutrition Knowledge among Young People with Orthorexic Tendencies. Nutrients, 14(20), Article 20. https://doi.org/10.3390/nu14204333
Reason for exclusion: ORTO cut-off point different than < 40 or < 35.
Kaźmierczak-Wojtaś, N., Niedzielski, A., & Drozd, M. (2022). Orthorexic tendencies and the structure of achievement motivation in young people in Poland. Nutrition, 103–104, 111795. https://doi.org/10.1016/j.nut.2022.111795
Reason for exclusion: ORTO cut-off point different than < 40 or < 35.
Kaźmierczak-Wojtaś, N., Patryn, R., Zagaja, A., Drozd, M., & Niedzielski, A. (2021). Prevalence and Characteristics of Orthorectic Disorders in Adolescence and Young People: Polish Preliminary Studies. Nutrients, 13(5), Article 5. https://doi.org/10.3390/nu13051568
Reason for exclusion: ORTO cut-off point different than < 40 or < 35.
Khalil, J., Boutros, S., Kheir, N., Kassem, M., Salameh, P., Sacre, H., Akel, M., Obeid, S., & Hallit, S. (2022). Eating disorders and their relationship with menopausal phases among a sample of middle-aged Lebanese women. BMC Women’s Health, 22(1), 153. https://doi.org/10.1186/s12905-022-01738-6
Reason for exclusion: Proportion of disordered eating not shown.

Li, W.-L., Tan, S.-X., Ouyang, R.-Q., Cui, Y.-F., Ma, J.-R., Cheng, C., Mu, Y.-J., Zhang, S.-W., Zheng, L., Xiong, P., Ni, W.-Z., Li, L.-Y., Fan, L.-N., Luo, Y.-M., Yu, Y.-L., Wang, Z.-M., Ding, F., Pan, Q.-F., Jiang, A.-Y., … Zeng, F.-F. (2022). Translation and validation of the Chinese version of the orthorexia nervosa assessment questionnaires among college students. Eating and Weight Disorders: EWD, 27(8), 3389–3398. https://doi.org/10.1007/s40519-022-01469-5
Reason for exclusion: Proportion of disordered eating not shown.
Łucka, I., Janikowska-Hołoweńko, D., Domarecki, P., Plenikowska-Ślusarz, T., & Domarecka, M. (2019). Orthorexia nervosa—A separate clinical entity, a part of eating disorder spectrum or another manifestation of obsessive-compulsive disorder? Psychiatria Polska, 53(2), 371–382. https://doi.org/10.12740/PP/OnlineFirst/85729
Reason for exclusion: Duplicated data.
MacPhail, D. C. G., & Oberle, C. D. (2022). Seeing Shred: Differences in muscle dysmorphia, orthorexia nervosa, depression, and obsessive-compulsive tendencies among groups of weightlifting athletes. Performance Enhancement & Health, 10(1), 100213. https://doi.org/10.1016/j.peh.2021.100213
Reason for exclusion: No ORTO-15 used.
Mavrandrea, P., & Gonidakis, F. (2022). Exercise dependence and orthorexia nervosa in Crossfit: Exploring the role of perfectionism. Current Psychology (New Brunswick, N.J.), 1–9. https://doi.org/10.1007/s12144-022-03585-y
Reason for exclusion: ORTO cut-off point different than < 40 or < 35.
Meule, A., Holzapfel, C., Brandl, B., Greetfeld, M., Hessler-Kaufmann, J. B., Skurk, T., Quadflieg, N., Schlegl, S., Hauner, H., & Voderholzer, U. (2020). Measuring orthorexia nervosa: A comparison of four self-report questionnaires. Appetite, 146, 104512. https://doi.org/10.1016/j.appet.2019.104512
Reason for exclusion: Proportion of disordered eating not shown.
Missbach, B., Hinterbuchinger, B., Dreiseitl, V., Zellhofer, S., Kurz, C., & König, J. (2015). When Eating Right, Is Measured Wrong! A Validation and Critical Examination of the ORTO-15 Questionnaire in German. PloS One, 10(8), e0135772. https://doi.org/10.1371/journal.pone.0135772
Reason for exclusion: ORTO cut-off point different than < 40 or < 35.
Moller, S., Apputhurai, P., & Knowles, S. R. (2019). Confirmatory factor analyses of the ORTO 15-, 11- and 9-item scales and recommendations for suggested cut-off scores. Eating and Weight Disorders: EWD, 24(1), 21–28. https://doi.org/10.1007/s40519-018-0515-0
Reason for exclusion: ORTO cut-off point different than < 40 or < 35.
Novara, C., Pardini, S., Visioli, F., & Meda, N. (2022). Orthorexia nervosa and dieting in a non-clinical sample: A prospective study. Eating and Weight Disorders: EWD, 27(6), 2081–2093. https://doi.org/10.1007/s40519-021-01353-8
Reason for exclusion: No ORTO-15 used.
Oberle, C. D., Marcell, H. S., & Noebel, N. A. (2022). Orthorexia nervosa and substance use for the purposes of weight control, conformity, and emotional coping. Eating and Weight Disorders: EWD, 27(2), 553–561. https://doi.org/10.1007/s40519-021-01190-9
Reason for exclusion: No ORTO-15 used.
Özgen, C., Köse, H., & Reyhan, S. (2021). Relationship between Exercise Addiction, Orthorexia Nervosa, and Sports Supplement Attitude in Turkish Fitness Participants. Progress in Nutrition, 23(4), Article 4. https://doi.org/10.23751/pn.v23i4.12482
Reason for exclusion: No ORTO-15 used.
Parisi, L., La Grutta, S., BAIDO, R., Epifanio, M. S., Carotenuto, M., Operto, F. F., TESTA, D., Vetri, L., & ROCCELLA, M. (2022). Bigorexia and orthorexia: An indissoluble relationship? Minerva Psychiatry, 62. https://doi.org/10.23736/S2724-6612.20.02095-6
Reason for exclusion: Excluded due to data unavailability.

Parra-Fernandez, M. L., Rodríguez-Cano, T., Onieva-Zafra, M. D., Perez-Haro, M. J., Casero-Alonso, V., Muñoz Camargo, J. C., & Notario-Pacheco, B. (2018). Adaptation and validation of the Spanish version of the ORTO-15 questionnaire for the diagnosis of orthorexia nervosa. PloS One, 13(1), e0190722. https://doi.org/10.1371/journal.pone.0190722
Reason for exclusion: ORTO cut-off point different than < 40 or < 35.
Plichta, M., & Jezewska-Zychowicz, M. (2020). Orthorexic Tendency and Eating Disorders Symptoms in Polish Students: Examining Differences in Eating Behaviors. Nutrients, 12(1), 218. https://doi.org/10.3390/nu12010218
Reason for exclusion: Duplicated data.
Plichta, M., Jezewska-Zychowicz, M., & Gębski, J. (2019). Orthorexic Tendency in Polish Students: Exploring Association with Dietary Patterns, Body Satisfaction and Weight. Nutrients, 11(1), 100. https://doi.org/10.3390/nu11010100
Reason for exclusion: Duplicated data.
Plichta, M., Jezewska-Zychowicz, M., & Małachowska, A. (2020). Relationship between Psychosocial Impairment, Food Choice Motives, and Orthorexic Behaviors among Polish Adults. Nutrients, 12(5), 1218. https://doi.org/10.3390/nu12051218
Reason for exclusion: Proportion of disordered eating not shown.
Pratt, V. B., Madigan, D. J., & Hill, A. P. (2022). Perfectionistic self-presentation and orthorexia in exercisers. Eating and Weight Disorders: EWD, 27(3), 1217–1221. https://doi.org/10.1007/s40519-021-01262-w
Reason for exclusion: Proportion of disordered eating not shown.
Rodgers, R. F., White, M., & Berry, R. (2021). Orthorexia nervosa, intuitive eating, and eating competence in female and male college students. Eating and Weight Disorders: EWD, 26(8), 2625–2632. https://doi.org/10.1007/s40519-020-01054-8
Reason for exclusion: ORTO cut-off point different than < 40 or < 35.
Rogoza, R., & Donini, L. M. (2021). Introducing ORTO-R: A revision of ORTO-15 : Based on the re-assessment of original data. Eating and Weight Disorders: EWD, 26(3), 887–895. https://doi.org/10.1007/s40519-020-00924-5
Reason for exclusion: Proportion of disordered eating not shown.
Roncero, M., Barrada, J. R., & Perpiñá, C. (2017). Measuring Orthorexia Nervosa: Psychometric Limitations of the ORTO-15. The Spanish Journal of Psychology, 20, E41. https://doi.org/10.1017/sjp.2017.36
Reason for exclusion: Proportion of disordered eating not shown.
Sanlier, N., Pehlivan, M., Sabuncular, G., Bakan, S., & Isguzar, Y. (2018). Determining the relationship between body mass index, healthy lifestyle behaviors and social appearance anxiety. Ecology of Food and Nutrition, 57(2), 124–139. https://doi.org/10.1080/03670244.2017.1419343
Reason for exclusion: Proportion of disordered eating not shown.
Segura-García, C., Papaianni, M. C., Caglioti, F., Procopio, L., Nisticò, C. G., Bombardiere, L., Ammendolia, A., Rizza, P., De Fazio, P., & Capranica, L. (2012). Orthorexia nervosa: A frequent eating disordered behavior in athletes. Eating and Weight Disorders: EWD, 17(4), e226-233. https://doi.org/10.3275/8272
Reason for exclusion: Proportion of disordered eating not shown.
Sfeir, E., Haddad, C., Salameh, P., Sacre, H., Hallit, R., Akel, M., Honein, K., Akiki, M., Kheir, N., Obeid, S., & Hallit, S. (2021). Binge eating, orthorexia nervosa, restrained eating, and quality of life: A population study in Lebanon. Eating and Weight Disorders: EWD, 26(1), 145–158. https://doi.org/10.1007/s40519-019-00831-4
Reason for exclusion: Proportion of disordered eating not shown.

Sifakaki M, Gkiouras K, Lindqvist HM, Marakis G, Petropoulou A, Donini LM, et al. Orthorexia Nervosa Practices in Rheumatoid Arthritis: The DORA Study. Nutrients. 2023 Jan 31;15(3):713
Reason for exclusion: Proportion of disordered eating not shown.
Tarı Selçuk, K., & Çevik, C. (2020). Use of dietary supplements among nursing students in Turkey in the last 12 months and its relation with orthorexia nervosa. Perspectives in Psychiatric Care, 56(4), 885–893. https://doi.org/10.1111/ppc.12507
Reason for exclusion: ORTO cut-off point different than < 40 or < 35.
Tóth-Király, I., Gajdos, P., Román, N., Vass, N., & Rigó, A. (2021). The associations between orthorexia nervosa and the sociocultural attitudes: The mediating role of basic psychological needs and health anxiety. Eating and Weight Disorders: EWD, 26(1), 125–134. https://doi.org/10.1007/s40519-019-00826-1
Reason for exclusion: ORTO cut-off point different than < 40 or < 35.
Toti, E., Cavedon, V., Raguzzini, A., Fedullo, A. L., Milanese, C., Bernardi, E., Bellito, S., Bernardi, M., Sciarra, T., & Peluso, I. (2022). Dietary Intakes and Food Habits of Wheelchair Basketball Athletes Compared to Gym Attendees and Individuals who do not Practice Sport Activity. Endocrine, Metabolic & Immune Disorders Drug Targets, 22(1), 38–48. https://doi.org/10.2174/1871530321666210208213046
Reason for exclusion: Proportion of disordered eating not shown.
Tremelling, K., Sandon, L., Vega, G. L., & McAdams, C. J. (2017). Orthorexia Nervosa and Eating Disorder Symptoms in Registered Dietitian Nutritionists in the United States. Journal of the Academy of Nutrition and Dietetics, 117(10), 1612–1617. https://doi.org/10.1016/j.jand.2017.05.001
Reason for exclusion: Subjects with eating disorder.
Valente, M., Syurina, E. V., Muftugil-Yalcin, S., & Cesuroglu, T. (2020). "Keep Yourself Alive": From Healthy Eating to Progression to Orthorexia Nervosa A Mixed Methods Study among Young Women in the Netherlands. Ecology of Food and Nutrition, 59(6), 578–597. https://doi.org/10.1080/03670244.2020.1755279
Reason for exclusion: Proportion of disordered eating not shown.
White, M., Berry, R., & Rodgers, R. F. (2020). Body image and body change behaviors associated with orthorexia symptoms in males. Body Image, 34, 46–50. https://doi.org/10.1016/j.bodyim.2020.05.003
Reason for exclusion: ORTO cut-off point different than < 40 or < 35.
Worsfold, K. A., & Sheffield, J. K. (2021). Practitioner eating disorder detection: The influence of health mindset, thin-ideal internalization, orthorexia and gender role. Early Intervention in Psychiatry, 15(2), 296–305. https://doi.org/10.1111/eip.12940
Reason for exclusion: ORTO cut-off point different than < 40 or < 35.
Yakın, E., Obeid, S., Fekih-Romdhane, F., Soufia, M., Sawma, T., Samaha, S., Mhanna, M., Azzi, R., Mina, A., & Hallit, S. (2022). "In-between orthorexia" profile: The co-occurrence of pathological and healthy orthorexia among male and female non-clinical adolescents. Journal of Eating Disorders, 10(1), 155. https://doi.org/10.1186/s40337-022-00673-z
Reason for exclusion: No ORTO-15 used.
Yakın, E., Raynal, P., & Chabrol, H. (2021). Distinguishing orthorexic behaviors from eating disordered and obsessive-compulsive behaviors: A typological study. Eating and Weight Disorders: EWD, 26(6), 2011–2019. https://doi.org/10.1007/s40519-020-01037-9
Reason for exclusion: No ORTO-15 used.
Yazkan, G. (2021). The relationship between orthorexia nervosa tendencies and OCD symptoms in healthcare professionals. Journal of Psychiatric Nursing. https://doi.org/10.14744/phd.2021.87369
Reason for exclusion: Proportion of disordered eating not shown.
Yilmaz, F. C. (2023). Orthorexia and eating attitudes in health sciences students. Nigerian Journal of Clinical Practice, 26(4), 502-507. https://doi.org/10.4103/njcp.njcp_507_20

Reason for exclusion: Proportion of disordered eating not shown.
Zakhour, M., Haddad, C., Sacre, H., Tarabay, C., Zeidan, R. K., Akel, M., Hallit, R., Kheir, N., Obeid, S., Salameh, P., & Hallit, S. (2021). Differences in the Associations between Body Dissatisfaction and Eating Outcomes by Gender? A Lebanese Population Study. Revue D'epidemiologie Et De Sante Publique, 69(3), 134–144. https://doi.org/10.1016/j.respe.2021.02.003
Reason for exclusion: Proportion of disordered eating not shown.
Zhou, X., Schneider, S. C., Cepeda, S. L., & Storch, E. A. (2020). Orthorexia Nervosa in China: An Exploration of Phenomenology and Clinical Correlates Among University Students. Journal of Cognitive Psychotherapy, 34(3), 225–241. https://doi.org/10.1891/JCPSY-D-19-00027
Reason for exclusion: Excluded due to data unavailability.

Table S3. Characteristics of the studies using the <35 cut-off point (N=34).

Reference	Year	Country	Study design	Total (n)	Women (%)	Age (mean)
Abdullah et al.	2020	Jordan	Cross-sectional	421	69.8	23.9
Aiello et al.	2022	Spain and Italy	Cross-sectional	160	57.5	24.1
Almeida et al.	2018	Portugal	Cross-sectional	193	58.5	32.8
Bert et al.	2019	Italy	Cross-sectional	549	25.3	26.7
Bo et al.	2014	Italy	Cross-sectional	440	54.1	19.8
Carpita et al.	2021	Italy	Cross-sectional	2426	65.5	26.9
Dell'Osso et al.	2016	Italy	Cross-sectional	2826	40.6	28.9
Donini et al.	2005	Italy	Cross-sectional	514	Not reported	Not reported
Dunn et al.	2017	USA	Cross-sectional	275	66	21.7
Gonidakis et al.	2020	Greece	Cross-sectional	120	Not reported	Not reported
Gorrasi et al.	2020	Italy	Cross-sectional	918	54.8	20.2
Guglielmetti et al.	2022	Italy	Cross-sectional	671	53.9	21.0
Gwioździk et al.	2022	Poland	Cross-sectional	420	100	24.0
Heiss et al.	2019	Not reported	Cross-sectional	381	80.8	31.0
Herranz Valera et al.	2014	Spain	Cross-sectional	136	65.4	36.7
Hyrnik et al.	2016	Poland	Cross-sectional	1899	52.2	17.4
Jerez et al.	2015	Chile	Cross-sectional	210	45.9	16.7
Karniej et al.	2023	Poland and Spain	Cross-sectional	394	0	38.1
Kujawowicz et al.	2022	Poland	Cross-sectional	123	100	34.0
Labossière & Thibault	2019	Canada	Cross-sectional	133	71.4	21.3
Lorenzon et al.	2020	Brazil	Cross-sectional	480	56.7	35.3
Łucka et al.	2019	Poland	Cross-sectional	864	69.3	19.8
Maghetti et al.	2015	Italy	Cross-sectional	517	53.8	Not reported
Malmborg et al.	2017	Sweden	Cross-sectional	207	56.5	22.8
Martinovic et al.	2022	Croatia	Cross-sectional	300	45	22.4
Plichta & Jezewska-Zychowicz	2019	Poland	Cross-sectional	1120	70.4	21.4
Raguzzini et al.	2021	Italy	Cross-sectional	56	51.8	61.7
Ramacciotti et al.	2011	Italy	Cross-sectional	177	63.8	38.0
Reynolds	2018	Australia	Cross-sectional	92	79.3	24.6
Stochel et al.	2015	Poland	Cross-sectional	399	53.4	16.9
Surała et al.	2020	Not reported	Cross-sectional	273	45.8	20.9
Turner & Lefevre	2017	Not reported	Cross-sectional	680	100	24.7
Uriegas et al.	2021	USA	Cross-sectional	1090	69.4	19.6
Voglino et al.	2021	Italy	Cross-sectional	240	68.8	44.0

Table S4. Characteristics of the studies included using the <40 cut-off point (N=64).

Reference	Year	Country	Study design	Total (n)	Women (%)	Age (mean)
Abdullah et al.	2020	Jordan	Cross-sectional	421	69.8	23.9
Aiello et al.	2022	Spain and Italy	Cross-sectional	160	57.5	24.1
Aksoydan & Camci	2009	Turkey	Cross-sectional	94	58.5	33.2
Albery et al.	2020	United Kingdom	Experimental study	80	75	29.4
Almeida et al.	2018	Portugal	Cross-sectional	193	58.5	32.8
Asil & Sürücüoğlu	2015	Turkey	Cross-sectional	117	86.3	34.0
Athanasaki et al.	2023	Greece	Cross-sectional	96	95.8	23.4
Bosi et al.	2007	Turkey	Cross-sectional	315	53.1	27.2
Bergonzi & Massarollo	2022	Brazil	Cross-sectional	61	95.1	23.0
Bert et al.	2019	Italy	Cross-sectional	549	25.3	26.7
Bién & Pieczykolan	2017	Poland	Cross-sectional	280	100	Not reported
Clifford & Blyth	2019	United Kingdom	Case-control	215	65.6	21.0
Cosentino et al.	2023	Italy	Cross-sectional	88	Not reported	40.0
De Marchi & Baratto	2018	Brazil	Cross-sectional	82	93.9	21.0
de Souza & Rodrigues	2014	Brazil	Cross-sectional	150	100	23.2
Demir & Bayram	2022	Turkey	Cross-sectional	310	65.8	31.8
Demirer & Yardımcı	2023	Turkey	Cross-sectional	197	53.3	30.6
Donini et al.	2005	Italy	Cross-sectional	514	NR	Not reported
Dunn et al.	2017	USA	Cross-sectional	275	66	21.7
Elias et al.	2022	Brazil	Cross-sectional	246	43.1	Not reported
Erdogan et al.	2022	Turkey	Cross-sectional	159	100	33.2
Ermumcu & Tek	2018	Turkey	Cross-sectional	132	100	31.7
Farchakh et al.	2019	Lebanon	Cross-sectional	627	50.4	21.8
Freire et al.	2020	Brazil	Cross-sectional	60	63.3	26.6
Gonidakis et al.	2020	Greece	Cross-sectional	120	Not reported	Not reported
Grajek et al.	2022	Poland	Cross-sectional	290	60.0	26.0
Gubiec et al.	2015	Poland	Cross-sectional	155	90.3	22.1
Haddad et al.	2019	Lebanon	Cross-sectional	589	66.5	27.6
Hamid et al.	2018	Malaysia	Cross-sectional	138	88.4	21.8
Hayles et al.	2017	USA	Cross-sectional	404	82.7	20.7
Heiss et al.	2019	Not reported	Cross-sectional	381	80.8	31.0
Herranz Valera et al.	2014	Spain	Cross-sectional	136	65.4	36.7
Karadağ et al.	2016	Turkey	Cross-sectional	750	50.0	25.8
Koven & Senbonmatsu	2013	USA	Cross-sectional	100	79	19.3

Kujawowicz et al.	2022	Poland	Cross-sectional	123	100	34.0
Lemos et al.	2018	Brazil	Cross-sectional	95	100	20.5
Lorenzon et al.	2020	Brazil	Cross-sectional	430	56.7	35.3
Łucka et al.	2019	Poland	Cross-sectional	864	69.3	19.8
Maghetti et al.	2015	Italy	Cross-sectional	517	53.8	Not reported
Malmborg et al.	2017	Sweden	Cross-sectional	207	56.5	22.8
Mitrofanova et al. A	2021	Greece	Cross-sectional	10	80	28.4
Mitrofanova et al. B	2021	United Kingdom	Cross-sectional	50	60	34.0
Penaforte et al.	2017	Brazil	Cross-sectional	141	90.8	21.5
Plichta & Jezewska-Zychowicz	2019	Poland	Cross-sectional	1120	70.4	21.4
Raguzzini et al.	2021	Italy	Cross-sectional	56	51.8	61.7
Ramacciotti et al.	2011	Italy	Cross-sectional	177	63.8	38.0
Reynolds	2018	Australia	Cross-sectional	92	79.3	24.6
Rizzieri et al.	2019	Brazil	Cross-sectional	65	55.4	29.9
Sampaio et al.	2022	Brazil	Cross-sectional	150	74.7	Not reported
Sanlier et al.	2016	Turkey	Cross-sectional	900	58	20.4
Stochel et al.	2015	Poland	Cross-sectional	399	53.4	16.9
Sünbül & Bayrak	2021	Turkey	Cross-sectional	580	43.1	20.9
Surała et al.	2020	Not reported	Cross-sectional	273	45.8	20.9
Tocchetto et al.	2018	Brazil	Cross-sectional	50	46.0	23.5
Turner & Lefevre	2017	Not reported	Cross-sectional	680	100	24.7
Uriegas et al.	2021	USA	Cross-sectional	1090	69.4	19.6
Vaccari et al.	2021	Italy	Cross-sectional	236	57.2	34.5
Varga et al.	2014	Hungary	Cross-sectional	810	89.4	32.4
Voglino et al.	2021	Italy	Cross-sectional	240	68.8	44.0
Yeşildemir & Tek	2022	Turkey	Cross-sectional	206	50	26.2
Yılmaz & Dundar	2022	Turkey	Cross-sectional	248	41.9	42.6
Yilmazel	2021	Turkey	Cross-sectional	969	63.9	21.4
Yilmazel & Bozdogan	2020	Turkey	Cross-sectional	420	46.2	43.4
Zańko et al.	2019	Poland	Cross-sectional	87	87	21.0

Table S5. Results of the quality assessment checklist for studies.

Reference	1	2	3	4	5	6	7	8	9	Total score	Summary on the overall risk of study bias
Abdullah et al. (2020)	0	0	1	0	0	0	0	0	0	1	Low risk
Aiello et al. (2022)	1	0	1	0	0	0	0	0	1	3	Low risk
Aksoydan & Camci (2009)	1	1	1	0	0	0	0	0	0	3	Low risk
Albery et al. (2020)	1	0	1	0	0	0	0	0	0	2	Low risk
Almeida et al. (2018)	1	0	1	0	0	0	0	0	0	2	Low risk
Asil & Sürücüoğlu (2015)	1	0	1	0	0	0	0	1	1	4	Moderate risk
Athanasaki et al. (2023)	1	0	1	0	0	0	0	0	0	2	Low risk
Bosi et al. (2007)	1	0	1	0	0	0	0	0	0	2	Low risk
Bergonzi & Massarollo (2022)	1	0	1	0	0	0	0	0	0	2	Low risk
Bert et al. (2019)	1	1	1	0	0	0	0	0	0	3	Low risk
Bién & Pieczykolan (2017)	1	1	1	0	0	0	0	0	0	3	Low risk
Bo et al. (2014)	1	1	1	0	0	0	0	0	0	3	Low risk
Carpita et al. (2021)	1	0	1	0	0	0	0	0	0	2	Low risk
Clifford & Blyth (2019)	1	1	1	0	0	0	0	0	0	3	Low risk
Cosentino et al. (2023)	1	0	1	0	0	0	0	0	0	2	Low risk
De Marchi & Baratto (2018)	1	0	1	0	0	0	0	0	0	2	Low risk
de Souza & Rodrigues (2014)	1	0	1	0	0	0	0	0	0	2	Low risk
Dell'Osso et al. (2016)	1	0	1	0	0	0	0	0	1	3	Low risk
Demir & Bayram (2022)	1	1	0	0	0	0	0	0	0	2	Low risk
Demirer & Yardımcı (2023)	1	0	1	0	0	0	0	0	0	2	Low risk
Donini et al. (2005)	1	0	0	0	0	0	0	0	0	1	Low risk
Dunn et al. (2017)	1	0	0	0	0	0	0	0	0	1	Low risk
Elias et al. (2022)	1	1	1	0	0	0	0	0	0	3	Low risk
Erdogan et al. (2022)	1	1	1	0	0	0	0	0	0	3	Low risk
Ermumcu & Tek (2018)	1	1	1	0	0	0	0	0	0	3	Low risk
Farchakh et al. (2019)	0	0	0	0	0	0	0	0	0	0	Low risk
Freire et al. (2020)	1	1	1	0	0	0	0	0	0	3	Low risk
Gorrasi et al. (2020)	1	1	1	0	0	0	0	0	0	3	Low risk
Gonidakis et al. (2020)	1	0	1	0	0	0	0	0	0	2	Low risk
Grajek et al. (2022)	1	1	1	0	0	0	0	0	0	3	Low risk
Gubiec et al. (2015)	1	0	1	0	0	0	0	0	0	2	Low risk
Guglielmetti et al. (2022)	1	1	1	0	0	0	0	0	0	3	Low risk
Gwioździk et al. (2022)	1	0	0	0	0	0	0	0	0	1	Low risk
Haddad et al. (2019)	0	0	0	0	0	0	0	0	0	0	Low risk
Hamid et al. (2018)	1	0	1	0	0	0	0	0	0	2	Low risk
Hayles et al. (2017)	1	0	1	0	0	0	0	0	0	2	Low risk
Heiss et al. (2019)	1	0	1	1	0	0	0	0	0	3	Low risk
Herranz Valera et al. (2014)	1	0	1	0	0	0	0	0	0	2	Low risk
Hyrnik et al. (2016)	1	0	0	1	0	0	0	0	0	2	Low risk
Jerez et al. (2015)	1	0	1	0	0	0	0	0	1	3	Low risk
Karadağ et al. (2016)	1	0	0	0	0	0	0	0	0	1	Low risk
Karniej et al. (2023)	0	0	1	0	0	0	0	0	0	1	Low risk
Koven & Senbonmatsu (2013)	1	0	1	0	0	0	0	0	0	2	Low risk
Kujawowicz et al. (2022)	1	0	1	0	0	0	0	0	0	2	Low risk
Labossière & Thibault (2019)	1	0	1	0	0	0	0	0	0	2	Low risk
Lemos et al. (2018)	1	0	1	0	0	0	0	0	0	2	Low risk
Lorenzon et al. (2020)	1	0	1	0	0	0	0	0	0	2	Low risk
Łucka et al. (2019)	1	0	0	0	0	0	0	0	0	1	Low risk
Maghetti et al. (2015)	0	0	0	0	0	0	0	0	0	0	Low risk
Malmborg et al. (2017)	1	1	1	0	0	0	0	0	0	3	Low risk
Martinovic et al. (2022)	0	0	0	0	0	0	0	0	0	0	Low risk
Mitrofanova et al. (2021) A	1	0	1	0	0	0	0	0	0	2	Low risk
Mitrofanova et al. (2021) B	1	1	1	0	0	0	0	1	0	4	Moderate risk
Penaforte et al. (2017)	1	0	1	0	0	0	0	0	0	2	Low risk
Plichta & Jezewska-Zychowicz (2019)	0	0	1	0	0	0	0	0	0	1	Low risk
Raguzzini et al. (2021)	1	0	1	0	0	0	0	0	0	2	Low risk
Ramacciotti et al. (2011)	0	0	0	0	0	0	0	0	0	0	Low risk
Reynolds (2018)	1	0	1	0	0	0	0	0	0	2	Low risk
Rizzieri et al. (2019)	1	0	1	0	0	0	0	0	0	2	Low risk
Sampaio et al. (2022)	1	1	1	0	0	0	0	0	0	3	Low risk

Sanlier et al. (2016)	1	0	1	0	0	0	0	0	0	2	Low risk
Stochel et al. (2015)	1	0	1	0	0	0	0	0	0	2	Low risk
Sünbül & Bayrak (2021)	1	0	0	0	0	0	0	0	0	1	Low risk
Surała et al. (2020)	1	1	1	0	0	0	0	0	0	3	Low risk
Tocchetto et al. (2018)	1	0	1	0	0	0	0	0	0	2	Low risk
Turner & Lefevre (2017)	1	0	1	0	0	0	0	0	0	2	Low risk
Uriegas et al. (2021)	1	0	0	0	0	0	0	0	0	1	Low risk
Vaccari et al. (2021)	0	1	1	0	0	0	0	0	0	2	Low risk
Varga et al. (2014)	1	0	1	0	0	0	0	0	0	2	Low risk
Voglino et al. (2021)	1	0	1	0	0	0	0	0	0	2	Low risk
Yeşildemir & Tek (2022)	1	0	1	0	0	0	0	0	0	2	Low risk
Yılmaz & Dundar (2022)	1	1	0	0	0	0	0	0	0	2	Low risk
Yilmazel & Bozdogan (2020)	1	0	0	0	0	0	0	0	0	1	Low risk
Yilmazel (2021)	1	0	1	0	0	0	0	0	0	2	Low risk
Zańko et al. (2019)	1	0	1	0	0	0	0	0	0	2	Low risk
